# The Canadian Cow-Calf Surveillance Network – productivity and health summary 2018 to 2022

**DOI:** 10.3389/fvets.2024.1392166

**Published:** 2024-04-10

**Authors:** Cheryl Waldner, M. Claire Windeyer, Marjolaine Rousseau, John Campbell

**Affiliations:** ^1^Large Animal Clinical Sciences, University of Saskatchewan, Saskatoon, SK, Canada; ^2^Faculty of Veterinary Medicine, University of Calgary, Calgary, AB, Canada; ^3^Département de Sciences Cliniques, Faculté de Médecine Vétérinaire, Université de Montréal, Saint-Hyacinthe, QC, Canada

**Keywords:** beef cow-calf, pregnancy loss, calving difficulty, calf disease, calf mortality

## Abstract

Cow-calf surveillance data provide critical information about changing herd demographics, productivity, health, and management as well as the opportunity to investigate the impact of differing management practices. A national cow-calf network was established to collect baseline information on herd health, production performance measures, and management. Questionnaires were used to collect information on herd attributes as well as data for the calving season, breeding, pregnancy testing, and weaning for herds from across Canada. From 2018 to 2022, a total of 565 calving record questionnaires, representing 110,658 calving female records from 171 herds were returned, along with 543 herd breeding to weaning questionnaires. Suggested benchmark values based on the 25th percentiles from Western and Eastern Canada were determined to be <5% for non-pregnancy, <2% for calf death from birth to 24 h, and < 2% for calf death from 24 h to weaning. Herds from Eastern Canada were more likely to report any assistance at calving (*p* < 0.001) than herds from Western Canada. Participating herds from the east had longer breeding (*p* < 0.001) and calving (*p* < 0.001) seasons than those from the west and were also more likely to use artificial insemination or embryo transfer (*p* < 0.001). Timing of calving season and use of large pastures for calving were not associated with mortality at birth. Herds that started calving before April were more likely to have calves die before 30 d than those that started calving later; however, this difference was not observed between 30 d and weaning. Herds that started to calve earlier were also more likely to report treating more calves for bovine respiratory disease, diarrhea, and navel or joint infections as well as to calf deaths from respiratory disease. Calves from herds in the east were more likely to be treated or die from diarrhea than from the west. Females from herds that started calving later were less likely to be pregnant. The unique longitudinal productivity and health data resulting from this network established a national baseline to address region-specific needs for knowledge translation and solutions to enhance productivity and support sustainability.

## 1 Introduction

Canada’s beef industry has seen changes associated with the consolidation of herds, an aging producer population (1), economic pressures (2), and threats from climate extremes (3). Animal health monitoring programs provide critical information to key members of industry, academia, and government to understand the impact of resulting changes in herd demographics, productivity, health, and management on the sustainability of the livestock industry. Surveillance systems also provide an opportunity for observational studies of the impact of management decisions and practices on privately owned beef operations and how these might vary temporally and geographically, particularly in a country such as Canada where the land used for raising beef is quite diverse.

In contrast to the surveillance model from the United States, where a very intensive system of questionnaires and on-farm sample collection has been completed every 10 years for the cow-calf industry (4), the Canadian cow-calf industry has focused on administering more frequent, less intensive questionnaires each year to accumulate longitudinal data from a smaller network of producers. The initial version of the surveillance network recruited herds from three Canadian prairie provinces (Alberta, Saskatchewan, and Manitoba) that were home to more than 80% of Canada’s cow-calf production. From 2013 to 2017, the Western Canadian Cow-Calf Surveillance Network (WCCCSN) maintained a longitudinal cohort study of >100 privately owned cow-calf herds and leveraged funding from a variety of sources to address numerous research questions, for example those related to antimicrobial use (AMU) and antimicrobial resistance (AMR) (5–7), prevention of infectious disease (8, 9), trace mineral deficiencies (10), animal welfare practices (11–13), trichomoniasis and bovine genital campylobacteriosis (14), parasite levels (15–17), and Johne’s disease (18).

The primary objective of the WCCCSN was to examine longitudinal data on reproductive performance and health in cow-calf herds and identify benchmarks for the most critical measures and important sources of differences among herds (19). Productivity data were collected following calving and weaning and pregnancy testing and summarized for important indices of herd performance. The most complete, reliable, and accurate performance metrics were identified as the percentage of females not pregnant when tested by a veterinarian, the percentage of calves dead within 24 h of birth, and the percentage of calves dead from 24 h to weaning.

This research network was, however, limited to the three prairie provinces and the need for a more national program recognizing region-specific needs was apparent (20). Herd size, management practices, regional forage options, and grazing practices differ greatly between Eastern and Western Canada (1, 20). A nationally based beef cattle health research network was necessary to provide crucial information for both industry and policy-makers. To meet this need, the Canadian Cow-Calf Surveillance Network (C3SN) was established and started recruiting herds in 2018.

The C3SN was also tasked with filling some of the data gaps recognized during the analysis of data from the WCCCSN. The WCCCSN had identified key performance benchmarks regarding reproductive performance and calf survival but questions remained regarding other outcomes of interest, including the frequency of assisted calvings, the number of calves treated for a disease, and regional differences in key breeding and calving management practices. The primary objective of this report is to describe the establishment of the C3SN and the resulting baseline information on herd productivity, health, and management collected between 2018 and 2022. The secondary objective is to compare key productivity outcomes across herds in different regions and with different timing of calving and intensity of calving management.

## 2 Approach/methodology

This study was approved and reviewed annually by the Animal Care Committee and Research Ethics Board (Protocol # 2014003) and the Behavioral Research Ethics Board (Beh-REB#309) at the University of Saskatchewan and the Comité d’éthique de la recherche en sciences et en santé (# CERSES-19-016-D) of the Université de Montréal. Modifications were submitted for approval as necessary to accommodate any changes in the research plan during the multi-year study. Informed consent was received from all study participants to share data summarized across herds.

### 2.1 Herd recruitment

Producers were initially recruited to the network in mid- to late 2018 through veterinarians, social media, provincial beef associations, and fellow producers. Targets for recruitment included operations that conducted pregnancy testing, maintained calving records, and had more than 40 breeding animals and access to email. Herd owners were contacted by phone so the study could be explained, interest assessed, and eligibility verified. Because herd recruitment was not completed until the end of 2018, only baseline management information was collected for 2018 at the time of enrollment together with the consent forms. The baseline questionnaire had been previously tested in similar herds (19).

### 2.2 Questionnaire design and distribution

Information on herd attributes, calving, health, and productivity data for each calving season and each breeding to weaning and pregnancy testing season were subsequently requested in June and December of each year from 2019 to 2022 using tools adapted from productivity questionnaires previously tested and proven with Canadian cow-calf producers (19). This questionnaire, originally designed in English, has been translated into French by a certified translator with experience in animal production, and then checked for clarity by one of the bilingual authors (MR). Copies of the bilingual questionnaire tools are available upon request to the corresponding author.

Briefly, the calving questionnaire assessed whether multiple groups were calving at different times and, if so, how the herd was divided up into different management groups, whether any heifers were calved, and if separate records were available for the heifers. If management groups differed in calving dates by more than 2 months, producers were asked to answer questions to reflect the majority of the herd. Questions asked independently for cows and heifers were related to the start and end of calving season and the numbers of females retained (expected to calve and open), aborted, and calved full-term. The next questions collected information on the numbers of assisted births and degree of assistance, twins, calves born dead or that died within 24 h, and calves that died from day 1 to 30. Producers were also asked to report the occurrence of hard pulls, defined for this study as a two-person assist or requiring a mechanical aid or calf jack. Producers were also asked about numbers of calves that were sick with, treated for, or died from diarrhea, respiratory disease, or navel or joint infections before 30 d of age. Finally, producers were asked about the number of animals of different types purchased between weaning and pasture turn out.

The breeding to weaning questionnaire similarly began with questions about whether any heifers were bred and if separate records were available. Questions about the breeding season for cows and heifers included the number of bulls used for natural service, females exposed to only natural service, females bred at least once with artificial insemination (AI) or embryo transfer, and the start and end dates for the breeding season. Producers were asked to report the numbers of animals purchased from the start of the breeding season through weaning by class. Questions about weaning included weaning dates, confirmation of the numbers of calves born alive, calves weaned, and calves that died between day 1 and weaning. Producers were also asked about numbers of calves that were sick with, treated for, or died from diarrhea, respiratory disease, or navel or joint infections between day 1 and weaning, and to describe any other disease outbreaks. Producers were asked to report whether all cows and bred heifers were pregnancy tested and, if not, which ones were checked. Finally, producers were asked what dates they pregnancy tested, the number of animals checked, and the number of animals not pregnant when tested.

Producers were provided with a binder at the start of the study containing these data collection tools and were reminded to submit the relevant pages each June and December. The binders with the questionnaires were provided instead of mailing new questionnaires at each time period due to the diversity in breeding and calving seasons within the network and to ensure producers had all appropriate forms in a timely manner to optimize data collection. Producers were given a modest honorarium for productivity data submitted after 2021 and then in 2023. Participants were considered to have dropped out of the study either after they communicated they wished to withdraw or when no responses to any questionnaires were received after at least one full production cycle.

### 2.3 Data management and statistical analysis

Data were entered as they were received into a commercial spreadsheet program and independently checked for accuracy. Subsequent logical checks were completed to verify the internal consistency of data among survey questions for each herd.

Because not all questions were answered on all submitted questionnaires, the number of observations varies from question to question, and as such the number of observations was reported for each outcome. For the calving questionnaire data to be eligible for analysis, the number of cows that calved had to be provided. For the breeding to weaning questionnaire, the minimum necessary data to evaluate any additional outcomes were the number of calves born alive. These values were deemed necessary as they were components of the denominator for most calving and weaning outcomes of interest.

Descriptive analysis included summarizing the distribution of results for each health and performance outcome of interest as well as management metrics calculated from the reported data. Data were summarized for the study as a whole (tables in the results section) and then separately for herds from Eastern and Western Canada (regional tables provided as Supplementary Files). Provinces in the west included British Columbia, Alberta, Saskatchewan, and Manitoba while the east was considered to include Ontario, Quebec, and the Maritime provinces.

Benchmarks for performance metrics describing losses were identified by first describing the 25th percentiles for key values of interest and then rounding them up to the nearest integer for herd data combined from both Western and Eastern Canada. Benchmarks were reported for the proportion of calves that died from birth to 24 h, for calves that died from 24 h to weaning, and for the proportion of cows that were not pregnant when examined by a veterinarian.

The first step in exploring the data was to examine differences in the frequency of productivity and health outcomes, as well as management practices of interest, between cows and heifers and between Eastern and Western Canada. Generalized estimating equations (GEE) was used to account for repeated measures on individual herds over time with an autoregressive correlation structure. Year of data collection and herd size were included in all models as potential confounders. The numerator was modeled as a fraction of the denominator using a binomial distribution and logit link function for outcomes reported as proportions (SAS version 9.4, Cary, NC). Where the outcome was a count, the outcome was modeled with a Poisson, normal or lognormal distribution as appropriate.

The second step in the analysis was to further investigate regional differences and follow up on previous work suggesting differences in outcomes based on time of breeding and calving and the intensity of herd management (19). This was intended to address the question of whether substantial differences in calving time and calving management intensity could account for observed east and west differences in productivity. Early calving herds from this cohort started calving in December through March while late calving herds started in April. The questionnaire results were classified based on whether the herd owner reported using any large pastures for calving vs. more intensively managed herds that did not report using any large pastures for calving and rather used only small paddocks, corrals, dry lots, barns, or covered sheds. Participants reported the area of pastures described as large on the questionnaire.

Important calving productivity and health outcomes were examined for differences among timing of calving, use of large pastures, and geographic region in a multivariable model after accounting for potential confounders as fixed effects including year, proportion of females calving that were heifers, proportion of the herd reported as purebred, and whether the total number of calving females was greater than 300. In cases where geographic region remained significant in the multivariable model, the region variable was temporarily removed in a second sensitivity analysis to investigate whether unmeasured regional management or environmental differences were moderating the impact of calving timing or large pasture use. If removing region substantially changed the result of either calving timing or large pasture use (difference > 20%), the second model was also reported. Group differences are reported as odds ratios (OR) with 95% confidence intervals (95%CI).

## 3 Results

### 3.1 Description of participating herds

Of the 180 producers that completed the 2018 baseline questionnaire at the time of enrollment, 171 returned the calving record questionnaire for 2019 including a total number of cows calved (Table 1) and are considered the initial participating herds for this study.

**Table 1:** Attributes reported on baseline questionnaire for 2018 for 171 Canadian cow-calf herds that subsequently submitted calving records in 2019.

Attribute	Percent	Attribute	Percent
Cow-calf operation is 100% commercial	63% (107/171)	Cow-calf records include:	
Production activities: Backgrounding	61% (105/171)	Calving date	98% (167/171)
Production activities: Stockers	30% (51/171)	Individual ID	93% (159/171)
Production activities: Feedlot	14% (24/171)	Calf’s ID linked to dam ID	92% (157/171)
Other livestock, Dairy	4% (7/171)	Wean weight	50% (85/171)
% Farm income from cow-calf operation		Birth weight	55% (94/171)
<25	6% (11/171)	Cull/death loss	88% (150/171)
25 to <50	21% (36/171)	Individual animal treatments	76% (130/171)
50 to <75	17% (29/171)	Describe herd-level drug use	59% (101/171)
75 to 100	54% (92/171)	Production records used for:	
% Family income from cow-calf operation		Culling	92% (157/171)
<25	25% (42/171)	Sire selection	49% (83/171)
25 to <50	22% (37/171)	Replacement heifer selection	88% (151/171)
50 to <75	23% (39/171)	Annual analysis of whole farm	47% (81/171)
75 to 100	27% (46/171)	Break evens	19% (33/171)
Any non-family employees	32% (54/171)	Production records kept on:	
Management decisions person <40 yrs	58% (100/171)	Paper	93% (159/171)
Management decisions person <30 yrs	25% (43/171)	Electronic spreadsheet	49% (84/171)
Education includes post-secondary	81% (138/171)	Computer-based program	30% (52/171)
Calving groups separated by >1 mo	27% (46/171)	Smartphone app	16% (28/171)
Separate calving groups, Age	5% (9/171)	Technologies / practices used in 2018:	
Separate calving groups, Breed	2% (4/171)	Hormone implant	36% (62/171)
Separate calving groups, Pure/Commercial	9% (15/171)	Ionophores	57% (97/171)
Primary area(s) used for calving:		Pain mitigation	74% (126/171)
Large pasture/open range	22% (37/171)	Nutritionist	53% (91/171)
Small grass paddock/pasture	27% (47/171)	Forage/ feed testing	73% (125/171)
Corrals/dry lot	40% (69/171)	Water testing	22% (38/171)
Barn/shed	27% (46/171)	Defined breeding season	47% (80/171)
Breeding soundness evaluation of all bulls	67% (115/171)	Estrus cycle	32% (55/171)
Breeding soundness evaluation of some bulls	7% (12/171)	Artificial insemination	42% (72/171)
Test bulls for trichomoniasis	7% (12/166)	Sexed semen	4% (7/171)
Test bulls for vibriosis	0% (0/166)	Embryo transfer	15% (26/171)
Cow/Heifers to communal grazing – all	4% (6/171)	Multi-sire breeding	57% (98/171)
Cow/Heifers to communal grazing – some	16% (27/171)	DNA parentage	23% (40/171)
Bulls sent to communal grazing	15% (26/170)	Genetic testing	14% (24/171)
Cow/Heifers bred at communal grazing	17% (28/168)	Individual female records	47% (80/171)

Most participating herds were 100% commercial and engaged in other beef industry activities in addition to cow-calf production such as backgrounding calves or pasturing stocker cattle (Table 1). For 71% of the herds, more than half of the overall farm income was the result of the cow-calf operation. However, the cow-calf operations varied more in terms of their contribution to total family income. Almost one in three herds had at least one non-family employee (Table 1).

Only one in four herds had at least one decision-maker under the age of 30. More than 80% of participants reported at least some post-secondary education (Table 1).

Corrals and dry lots were the most common primary calving locations reported in the baseline questionnaire (Table 1). Of the 22% of producers reporting using large pastures for calving, the median reported pasture size was 110 acres (25th percentile 43 acres, 75th percentile 315 acres). Two out of three participants reported breeding soundness evaluation testing all of their bulls, but very few reported testing their bulls for infectious disease (Table 1). At least some contact with other herds from grazing on community pastures was reported by 20% [(6 + 27)/171] of producers (Table 1).

Data reported in the baseline questionnaire were derived in part from calving records that included calving dates and individual animal identification in 98 and 93% of participating herds, respectively (Table 1). Disease information was based on individual animal treatment records in 76% of herds (Table 1). As a measure of biosecurity risk, animals purchased were documented between weaning and the start of breeding season (Supplementary Table S1) and between breeding and weaning (Supplementary Table S2).

### 3.2 Summary of calving records submitted from participating herds

A total of 565 herd calving record questionnaires were subsequently returned for the period from 2019 through 2022 (Table 2), representing 110,658 calving female records (93,355 cows and 17,303 heifers). More than 95% of producers calved at least one heifer, and between 93 and 96% of those provided separate annual records for heifers and cows (Table 2). For producers reported calving heifers and provided separate records, the number of heifers calved relative to total females calving varied from 13 to 17% depending on the year and location (Table 2).

**Table 2:** Attributes of Canadian cow-calf herds submitting annual herd calving records (*n* = 565) for the C3SN between 2019 and 2022.

Year data collected	2019	2020	2021	2022
Number of herds	*N* = 171	*N* = 152	*N* = 125	*N* = 117
Western Canada	66% (113/171)	66% (101/152)	68% (85/125)	68% (80/117)
British Columbia	5% (8/171)	5% (7/152)	5% (6/125)	4% (5/117)
Alberta	28% (48/171)	29% (44/152)	29% (36/125)	29% (34/117)
Saskatchewan	22% (37/171)	21% (32/152)	21% (26/125)	21% (25/117)
Manitoba	12% (20/171)	12% (18/152)	14% (17/125)	14% (16/117)
Eastern Canada	34% (58/171)	34% (51/152)	32% (40/125)	32% (37/117)
Ontario	15% (26/171)	16% (24/152)	17% (21/125)	17% (20/117)
Quebec	16% (28/171)	15% (23/152)	12% (15/125)	11% (13/117)
Maritime provinces	2% (4/171)	3% (4/152)	3% (4/125)	3% (4/117)
Sales of some seedstock	37% (64/171)	38% (57/152)	36% (45/125)	38% (44/117)
Western herds	33% (37/113)	33% (33/101)	33% (28/85)	33% (26/80)
Eastern herds	47% (27/58)	47% (24/51)	43% (17/40)	49% (18/37)
Number of cows calving (median, 5th & 95th percentile)	129 (35, 413)	121 (33, 425)	114 (33, 391)	110 (34, 325)
Western herds	177 (45, 446)	169 (52, 569)	157 (50, 469)	158 (42, 371)
Eastern herds	69 (29, 229)	66 (29, 207)	53 (27, 196)	62 (24, 192)
Number of heifers calving (median, 5th & 95th percentile)	25 (5, 96)	21 (4, 99)	20 (5, 89)	21 (5, 90)
Western herds	34 (10, 122)	33 (10, 113)	28 (9, 96)	30 (9, 101)
Eastern herds	11 (3, 46)	9 (2, 28)	10 (2, 22)	9 (4, 36)
Percent of total females calving that are heifers* (median, 5th & 95th percentile)	16% (7, 30%)	16% (7, 28%)	15% (7, 26%)	16% (8, 26%)
Western herds	17% (8, 36%)	17% (8, 34%)	16% (8, 26%)	16% (9, 27%)
Eastern herds	15% (6, 26%)	13% (5, 26%)	13% (5, 27%)	15% (6, 32%)
Do not calve any heifers	2% (4/171)	5% (7/152)	2% (3/125)	3% (3/117)
Reported number of heifers** calved separately from cows	93% (156/171–4)	94% (137/152–7)	96% (117/125–3)	96% (110/117–3)
Total number of cows with calving records	*n* = 28,967	*n* = 26,491	*n* = 20,021	*n* = 17,876
Total number of heifers with calving records	*n* = 5,716	*n* = 4,607	*n* = 3,616	*n* = 3,364

*Total number of heifers calving/total females calving where number heifers calving > 0.

**Denominator is total number of herds reporting any heifers (total herds providing calving records less herds that did not calve any heifers).

Of the 171 producers who provided calving questionnaires in 2019, 68% (117) continued to provide completed questionnaires through 2022 (Table 2). The proportion of herds completing the fourth year of data collection was similar in Eastern (64%, 37/58) and Western (71%, 80/113) Canada. While participant attrition occurred over the course of the study, characteristics of contributing herds remained relatively constant based on the proportion of commercial herds, herd size, and geographic distribution (Table 2).

Most herds from Eastern Canada were smaller than those from Western Canada (Table 2), with 10 producers smaller than the target herd size of 40 calving cows for at least one study year (8 Eastern Canada, 2 Western Canada); of these, two producers had <30 cows. The average number of cows (*p* < 0.001), heifers (p < 0.001), and total females (*p* < 0.001) calving for the herds from Western Canada (Supplementary Table S3a) was greater than for the herds from Eastern Canada (Supplementary Table S3b).

Sales of at least some seedstock were reported by 36–38% of producers providing calving records each year (Table 2). Participants were most likely to report purchasing bulls, replacement heifers, and cows (Supplementary Tables S1, S2). Producers were much less likely to report purchasing calves or cow-calf pairs.

### 3.3 Summary of productivity and health outcomes from calving records

The proportion of heifers that aborted (OR 1.7, 95%CI 1.3–2.1, *p* < 0.001) or had a calf that died within the first 24 h (OR 1.7, 95%CI 1.5–1.9, *p* < 0.001) was higher than for cows (Table 3). However, the number of heifers contributing to the calculation for each herd was also much smaller, resulting in the proportion of abortions and calf deaths being more variable amongst herds than for cows. The proportion of total females that aborted (*p* = 0.94) or had a calf that died within the first 24 h (*p* = 0.11) was not significantly different in Western Canada (Supplementary Table S3a) compared to Eastern Canada (Supplementary Table S3b) after accounting for year and herd size.

**Table 3:** Summary of pregnancy outcome indicators from Canadian cow-calf herds reported in submitted annual herd calving records (*n* = 565) for the C3SN between 2019 and 2022.

	Number females calving			Percent of pregnant cows that aborted			Percent of calves dead within 24 h			Percent of calving females with twins		
	Cows	Heifers	Total	Cows	Heifers	Total	Cows	Heifers	Total	Cows	Heifers	Total
Total herd records	*N* = 565	*N* = 520	*N* = 565	*N* = 559	*N* = 519	*N* = 559	*N* = 563	*N* = 523	*N* = 563	*N* = 564	*N* = 523	*N* = 564
Mean	165	33	196	1.6%	2.9%	1.8%	2.5%	3.9%	2.7%	3.1%	1.4%	2.9%
SD*	151	35	180	1.9%	9.3%	2.5%	2.3%	5.7%	2.2%	2.6%	3.3%	2.3%
2.5th percentile	27	3	32	0.0%	0.0%	0.0%	0.0%	0.0%	0.0%	0.0%	0.0%	0.0%
5th percentile	33	4	39	0.0%	0.0%	0.0%	0.0%	0.0%	0.0%	0.0%	0.0%	0.0%
25th percentile	65	11	78	0.0%	0.0%	0.5%	1.0%	0.0%	1.2%	1.3%	0.0%	1.3%
Median	120	22	138	1.1%	0.0%	1.3%	2.1%	1.7%	2.2%	2.6%	0.0%	2.4%
75th percentile	226	43	261	2.2%	3.1%	2.3%	3.4%	6.0%	3.8%	4.2%	1.3%	3.9%
95th percentile	407	97	490	4.7%	10.0%	4.7%	6.5%	14.3%	6.9%	7.9%	7.1%	7.1%
97.5th percentile	615	131	754	6.5%	16.7%	6.9%	8.3%	20.0%	8.3%	9.8%	9.1%	8.7%

Values for herds from Western and Eastern Canada are reported separately in Supplementary Tables S3a,b.

*Standard deviation.

In half of all herd responses, less than 5% of females were assisted at calving, and a median of only 1.5% of calving females required what was described as a hard pull (Table 4). No Caesarian section surgeries were reported in more than 75% of herd questionnaires. As expected, the proportion of heifers reported as requiring any assistance at calving (OR 4.1, 95%CI 3.1–5.4, *p* < 0.001), an easy pull (OR 3.4, 95%CI 2.7–4.3, *p* < 0.001), a hard pull (OR 4.2, 95%CI 3.2–5.4, *p* < 0.001), or surgery (OR 8.0, 95%CI 4.7–13.7, *p* < 0.001) was higher than for cows after accounting for year and herd size.

**Table 4:** Summary of calving difficulty outcomes from Canadian cow-calf herds reported in submitted annual herd calving records (*n* = 565) for the C3SN between 2019 and 2022.

	Percent of females calved assisted			Percent of females with easy pull*			Percent of females with hard pull**			Percent of females with caesarean section		
	Cows	Heifers	Total	Cows	Heifers	Total	Cows	Heifers	Total	Cows	Heifers	Total
Total herd records	*N* = 562	*N* = 523	*N* = 562	*N* = 559	*N* = 520	*N* = 559	*N* = 560	*N* = 521	*N* = 560	*N* = 558	*N* = 522	*N* = 558
Mean	5.4%	18.7%	7.4%	3.7%	10.3%	4.7%	1.9%	8.3%	2.8%	0.1%	0.7%	0.2%
SD***	7.7%	18.6%	8.6%	8.0%	12.7%	8.0%	3.0%	13.3%	3.7%	0.4%	2.0%	0.5%
2.5th percentile	0.0%	0.0%	0.0%	0.0%	0.0%	0.0%	0.0%	0.0%	0.0%	0.0%	0.0%	0.0%
5th percentile	0.0%	0.0%	0.0%	0.0%	0.0%	0.0%	0.0%	0.0%	0.0%	0.0%	0.0%	0.0%
25th percentile	1.1%	5.0%	2.2%	0.0%	0.0%	0.9%	0.0%	0.0%	0.5%	0.0%	0.0%	0.0%
Median	2.8%	13.3%	4.7%	1.4%	6.7%	2.5%	0.9%	4.0%	1.5%	0.0%	0.0%	0.0%
75th percentile	6.9%	26.5%	9.8%	4.0%	15.4%	5.7%	2.3%	10.8%	3.8%	0.0%	0.0%	0.0%
95th percentile	20.0%	53.5%	22.6%	13.0%	36.4%	13.8%	8.1%	33.3%	10.3%	0.8%	5.4%	1.4%
97.5th percentile	25.0%	66.7%	28.2%	19.6%	47.5%	21.6%	10.3%	44.4%	13.3%	1.4%	7.1%	2.0%

Values for herds from Western and Eastern Canada are reported separately in Supplementary Tables S4a,b.

*One person only assist.

**Two person assist or required mechanical aid (e.g., calf jack).

***Standard deviation.

Herds from Eastern Canada (Supplementary Table S4b) were more likely to report any assistance at calving (OR 2.7, 95%CI 1.8–4.1, p < 0.001), an easy pull (OR 3.1, 95%CI 1.8–5.4, *p* < 0.001), or a hard pull (OR 3.5, 95%CI 2.4–5.2, *p* < 0.001) than herds from Western Canada (Supplementary Table S4a) after accounting for year and herd size. The frequency of Caesarean section surgeries was not significantly different (*p* = 0.10) between regions.

Reported losses of calves from 24 h to 30 days of age were similar for cows and heifers (*p* = 0.20) and overall averaged 1.9% of calves born (Table 5); no significant difference was noted between Western (Supplementary Table S5a) and Eastern (Supplementary Table S5b) Canada after accounting for year and herd size (*p* = 0.11).

**Table 5:** Summary of calf sickness and death loss from 24 h to 30 days of age from Canadian cow-calf herds reported in submitted annual herd calving records (*n* = 565) for the C3SN between 2019 and 2022.

	Percent of calves dead 24 h to 30 d			Percent of calves reported treated with antibiotics 24 h to 30 d			Percent of calves dead with attributed cause 24 h to 30 d			
	Cows	Heifers	Total	Calf diarrhea	Respiratory disease	Navel or joint infection	Calf diarrhea	Respiratory disease	Navel or joint infection	Total
Total herd records	*N =* 562	*N =* 521	*N =* 562	*N =* 557	*N =* 558	*N =* 556	*N =* 558	*N =* 558	*N =* 558	*N =* 558
Mean	1.8%	2.2%	1.9%	3.9%	3.1%	2.2%	0.5%	0.4%	0.1%	1.0%
SD*	2.1%	6.0%	2.1%	8.1%	5.7%	4.0%	1.3%	1.3%	0.6%	2.1%
2.5th percentile	0.0%	0.0%	0.0%	0.0%	0.0%	0.0%	0.0%	0.0%	0.0%	0.0%
5th percentile	0.0%	0.0%	0.0%	0.0%	0.0%	0.0%	0.0%	0.0%	0.0%	0.0%
25th percentile	0.4%	0.0%	0.0%	0.0%	0.0%	0.0%	0.0%	0.0%	0.0%	0.0%
Median	1.3%	0.0%	1.2%	0.9%	0.7%	0.0%	0.0%	0.0%	0.0%	0.0%
75th percentile	2.6%	2.6%	4.3%	3.4%	2.3%	0.5%	0.3%	0.0%	1.2%	1.2%
95th percentile	5.6%	10.5%	16.8%	13.6%	10.1%	2.5%	1.9%	0.6%	4.0%	4.1%
97.5th percentile	6.6%	14.3%	23.6%	19.3%	14.3%	3.7%	2.5%	1.0%	5.2%	5.5%

Values for herds from Western and Eastern Canada are reported separately in Supplementary Tables S5a,b.

*Standard deviation.

The overall average proportion of calves reported as treated for diarrhea in the first 30 days of life was very similar to the proportion treated for respiratory disease (3.9 and 3.1%, respectively), but higher than that for navel or joint infection (2.2%) (Table 5). However, while the proportion of calves treated for respiratory disease (*p* = 0.14) or navel infection (*p* = 0.11) in the first 30 days was not significantly different between the east and west, the likelihood that a calf would be treated for diarrhea was significantly higher in herds from Eastern Canada (OR 3.1, 95%CI 1.9–5.1, *p* < 0.001) (Supplementary Tables S5a,b) after accounting for year and herd size.

The total proportion of all deaths attributed to diarrhea, respiratory disease, or navel or joint infection (mean = 1.0%) based on herd owner reports was just over half of the total reported death loss regardless of cause during the period from 2019 to 2022 (mean = 1.9%) (Table 5). No significant differences were noted in the death losses attributed to respiratory disease (*p* = 0.06) or calf diarrhea (*p* = 0.07) by the owners for herds located in Western and Eastern Canada (Supplementary Tables S5a,b) after accounting for year and herd size. However, the proportion of calves reported to have died from navel or joint infection was higher in herds from Eastern Canada than Western Canada (OR 4.4, 95%CI 1.7–11, *p* = 0.002) after accounting for year and herd size.

### 3.4 Summary of breeding management and pregnancy testing data submitted from participating herds

A total of 543 breeding to weaning questionnaires were returned for the period 2019 through 2022 (Table 6). One did not report females exposed to breeding, with the remaining 542 reporting 120,428 females exposed to breeding.

**Table 6:** Summary of seasonal timing of important production events for Canadian cow-calf herds reported in submitted annual herd calving records (*n* = 565) and calving to breeding records (*n* = 543) for the C3SN between 2019 and 2022.

	Breeding season start		Pregnancy testing	Calving season start		Weaning	
	Cows	Heifers		Cows	Heifers	Cows	Heifers
Western Canada							
Jan to Mar	7% (27/364)	9% (34/364)	4% (14/364)	69% (263/379)	74% (280/379)	2% (8/364)	2% (8/364)
Apr to Jun	63% (229/364)	64% (234/364)	1% (2/364)	26% (100/379)	22% (83/379)	0.3% (1/364)	0.3% (1/364)
Jul to Sep	30% (108/364)	21% (76/364)	10% (38/364)	0% (0/379)	0% (0/379)	12% (43/364)	11% (41/364)
Oct to Dec	0%	0%	80% (291/364)	4% (16/379)	4% (16/379)	85% (311/364)	80% (292/364)
Eastern Canada							
Jan to Mar	42% (76/179)	34% (61/179)	7% (12/179)	35% (65/186)	46% (85/186)	7% (13/179)	4% (8/179)
Apr to Jun	25% (44/179)	26% (46/179)	4% (8/179)	32% (60/186)	25% (47/186)	3% (5/179)	3% (5/179)
Jul to Sep	27% (49/179)	25% (45/179)	13% (24/179)	5% (10/186)	8% (14/186)	36% (64/179)	32% (57/179)
Oct to Dec	4% (8/179)	6% (11/179)	62% (111/179)	27% (51/186)	22% (40/186)	50% (90/179)	47% (84/179)

The enrolled herds from Western Canada were more likely than those from Eastern Canada to start the breeding season in the period from April to June, with most participants in Western Canada starting to calve from January to March (Table 6). Participating herds from Eastern Canada were more variable with respect to the timing of breeding and calving seasons.

All cows were pregnancy tested in more than three quarters of herd record submissions (Table 7). No difference was noted between Western and Eastern Canada in terms of the number of herds reporting complete pregnancy tests (*p* = 0.14). Those who reported testing some of the herd most frequently reported not testing cows they were planning to cull, or just testing heifers, second calvers, other females considered at high risk of being open, just testing cows located where they had suitable facilities, or just testing the purebred part of the herd. A small number of herds reported not testing in specific years due to weather conditions and other unplanned challenges.

**Table 7:** Summary of reproductive management for Canadian cow-calf herds reported in submitted annual herd calving records (*n* = 565) and breeding to weaning records (*n* = 543) for the C3SN between 2019 and 2022.

	Western Canada		Eastern Canada	
% herds breeding heifers ≥2 weeks earlier than cows*	% of annual herd reports 12% (42/364)	% not reported 5% (20/364)	% of annual herd reports 10% (18/179)	% not reported 10% (18/179)
Breeding season ≤ 63 days	cows: 32% (118/364)	heifers: 40% (144/364)	cows: 12% (22/179)	heifers: 15% (27/179)
Artificial insemination / embryo transfer Cows Heifers	% of herds reporting any30% (34/112)35% (39/112)	% of females in herds using any: mean (SD***)28% (24%)66% (32%)	% of herds reporting any59% (35/59)58% (34/59)	% of females in herds using any: mean (SD)44% (30%)75% (29%)
% pregnancy tested all females	85% (309/364)		78% (140/179)	
% pregnancy tested some females	11% (40/364)		19% (34/179)	
Length of calving season ** Cows Heifers	days: median (5th and 95th percentile)93 (65, 157)74 (47, 130)	% not reported2% (9/379)7% (26/379)	days: median (5th and 95th percentile)162 (67, 350)90 (34, 251)	% not reported2% (3/186)13% (24/186)
% herds heifer calving season ≥2 weeks earlier than cows	% of annual herd reports 15% (56/379)	% not reported 6% (22/379)	% of annual herd reports 9% (16/186)	% not reported 12% (22/186)

*Start of heifer breeding season less date start of cow breeding season.

**Date last full-term calf was born less date when first full-term calf was born to female bred in this herd.

***Standard deviation.

A relatively small percentage (10–12%) of herd records from both Eastern and Western Canada reported breeding start dates for their heifers that were more than 2 weeks ahead of the main cow herd (Table 7). The percentage of herd records that described starting breeding (*p* = 0.86) or calving (*p* = 0.43) of heifers more than 2 weeks before the cows did not differ between the east and west.

The size of herds providing breeding data varied greatly across the study as did the reported female to bull ratios (Table 8). The total number of females exposed to breeding was higher in Western Canada than in Eastern Canada (*p* < 0.001); however, the cow to bull ratio was not different in herds in the east vs. west (*p* = 0.51) (Supplementary Tables S6a,b).

**Table 8:** Summary of breeding stock and female to bull ratios for Canadian cow-calf herds reported in submitted annual herd breeding to weaning records (*n* = 543) for the C3SN between 2019 and 2022.

	Bulls bred to cows	Bulls bred to heifers	Cows exposed to breeding	Heifers exposed to breeding	Cow to bull ratio	Heifer to bull ratio
Total herd records	*N* = 542	*N* = 512	*N* = 542	*N* = 517	*N* = 535	*N* = 448
Mean	8	2	185	39	23	16
SD*	8	2	170	46	9	11
2.5th percentile	1	0	32	1	6	1
5th percentile	1	1	38	3	10	2
25th percentile	3	1	75	12	19	8
Median	6	1	130	25	22	15
75th percentile	10	2	249	51	27	22
95th percentile	21	5	430	122	38	33
97.5th percentile	34	6	699	160	42	37

Values for herds from Western and Eastern Canada are reported separately in Supplementary Tables S6a,b.

*Standard deviation.

Participating herds from Eastern Canada had longer breeding (*p* < 0.001) and calving (*p* < 0.001) seasons than those from Western Canada (Table 7 and Supplementary Tables S7a,b). Participants from Eastern Canada were also more likely to use AI or embryo transfer (*p* < 0.001).

Less than third of herd records described a breeding season length consistent with a defined breeding season of three cycles or less (Tables 7, 9). Herd owners from Western Canada were more likely than those from Eastern Canada to report a breeding season of 63 days or less for both cows (*p* = 0.001) and heifers (*p* = 0.008) (Supplementary Tables S7a,b). Only one herd record described a breeding season of two cycles or less for cows, while 8.8% (48/543) of reports from 22 different herds described breeding seasons of two cycles or less for heifers: 12% (45/364) of reports from 19 herds in the west and 1.7% (3/179) from 3 herds in the east.

**Table 9:** Summary of percentage of females bred using reproductive technologies and breeding season duration for Canadian cow-calf herds reported in submitted annual herd breeding to weaning records (*n* = 543) for the C3SN between 2019 and 2022.

	Percent of cows bred AI/ET	Percent of heifers bred AI/ET	Breeding season length cows (days)	Breeding season length heifers (days)
Total herd records	*N* = 542	*N* = 506	*N* = 519	*N* = 492
Mean	12.1%	23.6%	101	90
SD*	23.8%	37.5%	54	48
2.5th percentile	0%	0%	46	26
5th percentile	0%	0%	50	31
25th percentile	0%	0%	63	59
Median	0%	0%	83	77
75th percentile	11.6%	50%	122	110
95th percentile	70%	100%	212	184
97.5th percentile	85%	100%	251	212

*Standard deviation.

Values for herds from Western and Eastern Canada are reported separately in Supplementary Tables S7a,b.

Less than 50% of herds reported any use of AI, with participants reporting a higher proportion of AI use for heifers than for cows (*p* < 0.001) (Table 9). Participants from Eastern Canada reported a higher proportion of cows (*p* < 0.001) and heifers (*p* < 0.001) bred by AI than those from Western Canada (Supplementary Tables S7a,b).

The percentage of heifers that were not pregnant (mean = 9.8%, SD = 11.4%) when tested was more variable than for cows (mean = 7.7%, SD = 5.7%) and was also slightly higher on average than for cows (OR 1.8, 95%CI 1.4–2.2, *p* < 0.001) (Table 10). Herds from Western and Eastern Canada were not different in terms of the percentage of cows (*p* = 0.92) and heifers (*p* = 0.20) that were not pregnant at pregnancy testing (Supplementary Tables S8a,b).

**Table 10:** Summary of number of females pregnancy tested and percentage of tested females that were not pregnant for Canadian cow-calf herds reported in submitted annual herd breeding to weaning records (*n* = 543) for the C3SN between 2019 and 2022 (including all pregnancy testing data provided).

	Number of cows pregnancy tested	Number of heifers pregnancy tested	Number of all females pregnancy tested	Percent of cows not pregnant	Percent of heifers not pregnant	Percent of all females not pregnant
Total herd records	*N* = 507	*N* = 483	*N* = 507	*N* = 507	*N* = 483	*N* = 507
Mean	179	40	216	7.7%	9.8%	8.2%
SD*	172	45	209	5.7%	11.4%	5.7%
2.5th percentile	14	3	24	0.0%	0.0%	1.0%
5th percentile	27	5	40	1.1%	0.0%	1.7%
25th percentile	68	13	83	3.9%	0.0%	4.3%
Median	125	26	152	6.5%	6.9%	6.8%
75th percentile	240	54	286	10.0%	13.6%	10.5%
95th percentile	448	118	583	18.0%	33.3%	19.3%
97.5th percentile	736	155	819	20.4%	41.2%	22.2%

Values for herds from Western and Eastern Canada are reported separately in Supplementary Tables S8a,b.

*Standard deviation.

The initial data analysis included all submitted pregnancy testing data. A second summary was done including only data for which the herd owner reported that all females had been pregnancy tested (Supplementary Table S8c); however, not all herds provided separate data for cows and heifers. The median proportion of all females that were not pregnant was very similar between the herd records based on complete data (6.8%) and those that were not (6.9%) (Table 10 and Supplementary Table S8c).

### 3.5 Summary of weaning data submitted from participating herds

A total of 543 breeding to weaning questionnaires were returned for the period 2019 through 2022 (Table 6), representing 107,948 calves born alive. Of the 167 producers who provided breeding to weaning questionnaires in 2019, 65% (109) provided questionnaires through 2022.

Fewer herds provided records of calf death losses to weaning than for many of the other metrics considered in this study (Table 11). For the herds that reported separate numbers for heifers (mean = 5.5%, SD = 8.8%), the percentage of calves lost from 24 h to weaning was slightly higher and more variable across herds than for cows (mean = 3.9%, SD = 4.3%) (OR 1.4, 95%CI 1.2–1.6, *p* = 0.001). Herds from Eastern and Western Canada showed no difference in the percentage of calves that died from either cows (*p* = 0.35) or heifers (*p* = 0.62) between birth and weaning after accounting for herd size and year (Supplementary Tables S9a,b).

**Table 11:** Summary of calving death loss from 24 h to weaning from Canadian cow-calf herds reported in submitted breeding to weaning records (*n* = 543) for the C3SN between 2019 and 2022.

	Calves alive at 24 h of age			Percent of calves dead 24 h to weaning		
	Cows	Heifers	Total	Cows	Heifers	Total
Total herd records	*N* = 543	*N* = 518	*N* = 543	*N* = 542	*N* = 481	*N* = 542
Mean	170	30	199	3.9%	5.5%	4.1%
SD*	185	34	209	4.3%	8.8%	4.1%
2.5th percentile	29	0	34	0.0%	0.0%	0.0%
5th percentile	34	0	39	0.0%	0.0%	0.0%
25th percentile	68	9	79	1.5%	0.0%	1.7%
Median	120	19	138	2.9%	2.4%	3.1%
75th percentile	228	40	259	5.0%	8.0%	5.2%
95th percentile	424	92	488	11.2%	21.4%	11.5%
97.5th percentile	683	126	779	14.0%	28.6%	13.9%

Values for herds from Western and Eastern Canada are reported separately in Supplementary Tables S9a,b.

*Standard deviation.

Total death loss from 24 h to 30 days was higher than for 24 h to weaning for 11% (57/535) of the herd records available at both time points for the same year; for 50 of these 57 herd records, a similar error was noted for other treatment outcomes at weaning, suggesting the respondent had described calf losses from 30 days to weaning as compared to total losses from 24 h to weaning as instructed. For the remainder of the non-zero records that passed the logical check of the total death at weaning being greater than or equal to the death loss reported at 30 days, the death loss at 30 days averaged 44% (SD 31%) of that reported at weaning.

From the records provided after weaning, respiratory disease and diarrhea were the most common reasons for treating with antimicrobials between birth and weaning as well as the most common reasons for calf deaths attributed to a cause (Table 12). The frequency of treatment (*p* = 0.52) or death loss (*p* = 0.50) due to respiratory disease and the frequency of treatment (*p* = 0.43) or death loss (*p* = 0.79) due to navel or joint infection were not different between Western and Eastern Canada (Supplementary Tables S10a,b). However, herds from Eastern Canada reported a higher frequency of calf diarrhea (OR 3.5, 95%CI 2.1–5.8, *p* < 0.001) and a higher frequency of calves reported to have died from diarrhea (OR 2.2, 95%CI 1.3–3.6, *p* = 0.003) than herds from Western Canada. Less than half of all reported calf deaths from birth to weaning (Table 11) were attributed by participants to diarrhea, respiratory disease, or navel or joint infection (Table 12). No additional information was provided on other causes of death.

**Table 12:** Summary of calf treatment and death loss for diarrhea, respiratory disease, and navel or joint infection from 24 h to weaning from Canadian cow-calf herds reported in submitted breeding to weaning records (*n* = 543) for the C3SN between 2019 and 2022.

	Percent of calves reported treated with antibiotics 24 h to weaning			Percent of calves dead with attributed cause 24 h to weaning			
	Calf diarrhea	Respiratory disease	Navel or joint infection	Calf diarrhea	Respiratory disease	Navel or joint infection	Total
Total herd records	*N* = 541	*N* = 541	*N* = 541	*N* = 541	*N* = 541	*N* = 541	*N* = 541
Mean	4.2%	5.3%	2.1%	0.7%	0.7%	0.2%	1.6%
SD*	9.8%	12.6%	3.9%	1.9%	2.8%	0.9%	4.2%
2.5th percentile	0.0%	0.0%	0.0%	0.0%	0.0%	0.0%	0.0%
5th percentile	0.0%	0.0%	0.0%	0.0%	0.0%	0.0%	0.0%
25th percentile	0.0%	0.0%	0.0%	0.0%	0.0%	0.0%	0.0%
Median	1.0%	1.7%	0.6%	0.0%	0.0%	0.0%	0.5%
75th percentile	4.3%	5.0%	2.4%	0.6%	0.8%	0.0%	1.9%
95th percentile	16.9%	19.3%	9.2%	3.0%	2.7%	1.0%	5.7%
97.5th percentile	27.0%	31.0%	12.8%	5.0%	4.0%	1.9%	8.5%

Values for herds from Western and Eastern Canada are reported separately in Supplementary Tables S10a,b.

*Standard deviation.

### 3.6 Benchmark values for Western and Eastern Canada based on 2019 to 2022 data

The 25th percentile for calving death loss from birth to 24 h was 1.2% for the west and 1.3% for the east for cows and heifers combined (Supplementary Tables S3a,b). The 25th percentile for total calving death loss from 24 h to weaning was 1.6% for the west and 1.9% for the east (Supplementary Tables S9a,b). The 25th percentile for percentage of tested cows that were not pregnant when tested by a veterinarian was 4.5% for the west and 3.8% for the east (Supplementary Tables S8a,b). Based on these values, benchmarks of <2% were chosen for both death loss from birth to 24 h and from 24 h to weaning, and a benchmark of <5% was chosen for proportion not pregnant. These values were rounded up from the greater of the 25th percentile values to simplify reporting, but in all cases were also less than the respective medians (Supplementary Tables S3a,b, S8a,b, S9a,b).

### 3.7 Comparison of data from Western Canada 2019 to 2022 to data previously reported for 2014 to 2017

Reproductive indices for herds from Western Canada for 2019 to 2022 (this study) and 2014 to 2017 (previously reported (19) and reproduced with permission from open access source and corresponding author – CW) were compared (Supplementary Tables S11a,b). Individual years were also compared to highlight year-to-year variability in the results for Western Canada (Supplementary Table S12) and for Eastern Canada (Supplementary Table S13).

### 3.8 Differences in productivity and health outcomes based on calving records among regions and herd management strategies

Health and productivity outcomes more commonly varied based on timing of calving season and management intensity (Table 13) than across geographic region after accounting for year, percentage of heifers, percentage of purebred cattle, and herd size.

**Table 13:** Summary of multivariable associations [odds ratios (OR) with 95% confidence interval (95%CI)] comparing productivity outcomes among Canadian cow-calf herds based on geographic region and calving management reported with calving records for the C3SN between 2019 and 2022 (*n* = 565 herd reports).

Outcome	Earlier calving starts December to March vs. Later calving starts April to September			No large pastures used for calving vs. Calved on large pasture			East vs. West		
	OR	95%CI	*p* value	OR	95%CI	*p* value	OR	95%CI	*p* value
Abortions	1.08	0.79, 1.47	0.63	**0.69**	**0.49, 0.98**	**0.04**	0.96	0.76, 1.21	0.72
Calf dead before 24 h	1.21	0.94, 1.34	0.21	0.91	0.75, 1.12	0.38	1.23	0.98, 1.53	0.07
Calf dead from 24 h to 30 days	**1.69**	**1.25, 2.30**	**0.001**	0.71	0.50, 1.01	0.06	1.15	0.88, 1.50	0.31
Treated calf respiratory disease to 30 days	**5.79**	**3.29, 10.2**	**<0.001**	1.43	0.70, 2.92	0.33	1.30	0.81, 2.09	0.27
Treated calf diarrhea to 30 days	**2.90**	**1.75, 4.82**	**<0.001**	1.44	0.77, 2.68	0.25	**2.93**	**1.72, 4.93**	**<0.001**
Treated navel or joint infection to 30 days	**4.04**	**2.77, 5.89**	**<0.001**	**3.73**	**2.28, 6.09**	**<0.001**	1.68	0.99, 2.86	0.06
Dead calf respiratory disease to 30 days	**4.63**	**2.29, 9.39**	**<0.001**	**0.39**	**0.17, 0.87**	**0.02**	1.77	0.98, 3.20	0.06
Dead calf diarrhea to 30 days	1.60	0.80, 3.21	0.19	0.53	0.24, 1.19	0.12	1.37	0.78, 2.42	0.27
Dead navel or joint infection to 30 days	**2.82**	**1.25, 6.37**	**0.01**	1.82	0.82, 4.03	0.14	1.52	0.75, 3.08	0.25

All models adjusted for study year, percentage of heifers calved, percentage of animals reported as purebred, and whether there were > 300 calving females.Values in bold font indicate statistically significant differences.

More abortions were reported by herds that used large pastures for calving than for herds that did not (*p* = 0.04) (Table 13).

Calves born in herds that calved later were less likely to die from 24 h to 30 days than calves born in herds that calved early (*p* = 0.001) (Table 13). Calves born to herds that start calving later were also less likely to be recorded as treated for respiratory disease, diarrhea, and navel or joint infections in the first 30 days than calves born in herds that calve early (*p* < 0.001) (Table 13). Calves born on large pastures were less likely to be treated for navel infection in the first 30 days than calves from herds that did not use large pastures (*p* < 0.001) (Table 13).

Calves born later were less likely to die from respiratory disease in the first 30 days than those born later (*p* < 0.001) (Table 13). Calves born to herds that did not use large pastures were less likely to die from respiratory disease than calves from herds that did use large pastures (*p* = 0.02).

After accounting for all other risk factors, calves from herds in the east were more likely to die from diarrhea in the first 30 days than calves born to herds in the west (Table 13).

### 3.9 Differences in productivity and health outcomes based on breeding to weaning records among regions and herd management strategies

Health and productivity outcomes assessed at pregnancy testing and weaning were also more likely to vary by timing of calving season and management intensity rather than by region (Table 14) after accounting for year, percentage of heifers, percentage of purebred cattle, and herd size.

**Table 14:** Summary of multivariable associations [odds ratios (OR) with 95% confidence interval (95%CI)] comparing productivity outcomes among Canadian cow-calf herds based on geographic region and calving management reported with breeding to weaning records for the C3SN between 2019 and 2022 (*n* = 543 herd records).

Outcome	Earlier calving starts December to March vs. Later calving starts April to September			No large pastures used for calving vs. Calved on large pasture			East vs. West		
	OR	95%CI	*p* value	OR	95%CI	*p* value	OR	95%CI	*p* value
Calves dead from 24 h to weaning	1.23	0.89, 1.69	0.21	0.94	0.63, 1.38	0.74	1.08	0.86, 1.34	0.52
Treated calf respiratory disease to weaning	**2.51**	**1.45, 4.35**	**0.001**	1.46	0.73, 2.91	0.29	1.07	0.65, 1.78	0.79
Treated calf diarrhea to weaning	**2.04**	**1.11, 3.73**	**0.02**	1.76	0.86, 3.59	0.12	**3.67**	**2.09, 6.42**	**<0.001**
Treated navel or joint infection to weaning	**3.49**	**2.31, 5.26**	**<0.001**	**4.33**	**2.42, 7.73**	**<0.001**	1.59	0.91, 2.79	0.10
Dead respiratory disease to weaning	**2.72**	**1.45, 5.12**	**0.002**	0.62	0.29, 1.34	0.23	1.24	0.73, 2.12	0.43
Dead calf diarrhea to weaning	1.50	0.82, 2.72	0.19	0.77	0.38, 1.56	0.47	**2.02**	**1.16, 3.53**	**0.01**
Dead navel or joint infection to weaning	**2.62**	**1.44, 4.48**	**0.002**	2.00	0.87, 4.62	0.10	1.19	0.60, 2.34	0.62
Not pregnant at pregnancy testing	**0.75**	**0.62, 0.90**	**0.002**	0.86	0.71, 1.05	0.13	0.90	0.74, 1.09	0.26

All models adjusted for study year, percentage of heifers calved, percentage of animals reported as purebred, and whether there were > 300 calving females.Values in bold font indicate statistically significant differences.

The frequency of calf death from birth to weaning was not different among calving dates, pasture size, and geographic regions (Table 14).

The frequency of calves reported as treated for respiratory disease (*p* = 0.001), diarrhea (*p* = 0.02), or navel or joint infection (*p* < 0.001) from birth to weaning was higher in herds that calved early than in herds that calved later (Table 14). Calves from herds that calved earlier were also more likely to die from respiratory disease or navel or joint infection. Calves from herds that used larger pastures were less likely to be treated for navel or joint infection before weaning than calves from herds that did not use large pastures (*p* < 0.001).

Calves from herds in Eastern Canada were more likely to be treated for (*p* < 0.001) and die from (*p* = 0.01) diarrhea than herds from Western Canada after accounting for all other risk factors (Table 14).

Females from herds that calved later were less likely to be pregnant than those in herds that calved earlier (*p* = 0.002) (Table 14).

## 4 Discussion

This study provides the first national-level data collected for the Canadian cow-calf industry and the first multi-year or longitudinal data for herds outside the three prairie provinces of Manitoba, Saskatchewan, and Alberta. The previous WCCCSN only collected data from Western Canada and productivity data were focused on key pregnancy and calf survival outcomes at birth and to weaning (19). The primary calving and breeding to weaning data from the 2019 to 2022 C3SN includes several additional outcomes identified as potential gaps in the previous 2014 to 2017 study.

The four directly comparable metrics between the two networks were the cumulative incidences of abortion, death from birth to 24 h, death from 24 h to weaning, and non-pregnancy. Based on these metrics, achievable benchmark values for Canadian cow-calf herds were determined to be <5% for non-pregnancy, <2% for calves born to cows and heifers for death from birth to 24 h, and < 2% for cows and heifers for calf death from 24 h to weaning; these are very similar to those from the previous WCCCSN report (19). These benchmarks are based on multiple years of longitudinal data that can account for some of the year-to-year variability that can occur with single-year estimates of productivity.

The frequency of abortion reported in the current study was slightly higher than in the WCCCSN (19). The numbers from the C3SN are more similar to previous work in Western Canadian cow-calf herds where audited follow-up of individual animal breeding, pregnancy testing, and calving records was used to reduce the risk of underreporting (21). The proportion of calves that died from birth to 24 h of age was very similar to that reported in the WCCCSN (19) and was almost identical when only herds from Western Canada were used in the comparison. Reported calf deaths from 24 h to weaning were slightly higher on average in the current study than in the WCCCSN (19). Finally, the proportion of females that were not pregnant when tested was very similar between the two reports.

This study provides new multi-year data for Canadian cow-calf herds on assisted calvings. This type of productivity data may also have significant year-to-year variability within herds. The overall average of 7.4% of females requiring any assistance at calving and 2.8% reported as having a hard pull was similar to and possibly slightly better than previous values reported for more than 200 herds in Western Canada of 8.9 and 3.7%, respectively (22). Less than 3% of cows were reported as being assisted in half of the herd records submitted and less than 7% in 75% of herd records submitted. The numbers for all calving assistance metrics were significantly higher for heifers than for cows. The numbers of hard pulls were considerably less than for all assists, with less than 4% of heifers requiring more intensive intervention in half of herd records and less than 11% of heifers requiring more substantial intervention in less than 75% of herd records. Even as defined, the term hard pull was not that specific and was quite subjective regarding the amount of force necessary as many producers will use the calf jack even for relatively easy pulls to minimize the risk of injury to the operator and to facilitate an efficient delivery. Very few females (<1.5%) in less than 5% of herd records were reported as requiring surgical intervention at calving. Reports of assistance at calving, whether or not the pull was classified as being difficult, were more common from participating herd owners in Eastern vs. Western Canada.

The second identified gap the C3SN addressed was more specific data on early calf losses. Previous postmortem examination studies with information on the age of calf death between 24 h and weaning reported 68% of calf deaths in the first 30 days after calving (23, 24). Another more recent study based on questionnaire data also found that most calf death loss occurred within the first month (25). While the WCCCSN did not collect data on when calf deaths occurred from birth to weaning, the current study asked producers to report losses between 24 h and 30 days after birth, which was at or before the time most spring calving herds would have been moving their herds to summer pastures. These data were then compared to the values reported for calf death loss from birth to weaning where both calving and breeding to weaning records were available for the herd in the same year. The results would suggest a small group of producers misread the question at weaning and reported calf deaths after 30 days and before weaning. However, the remaining records passing all logical checks suggested that almost half of the death losses from 24 h to weaning were reported in the first 30 days.

The C3SN is also the first pan-Canadian study to collect longitudinal data on treatment for the most commonly reported calfhood diseases and reasons for AMU, in addition to death loss in cow-calf herds for both the first month of life and for the period from birth to weaning (26). Differences in treatments between cows and heifers were not requested due to concerns about whether producers would be able to easily provide those data. Only about half of all reported deaths for either the 24 h to 30 day or 24 h to weaning periods were attributed by herd owners to respiratory disease, diarrhea, or navel or joint infection. Our understanding of the reasons for death losses in cow-calf herds is lacking as the most recent study looking at causes of death based on post-mortem examination dates back to 2002 (24).

This study provided an opportunity to compare cow-calf herds from Eastern and Western Canada and help target future research and knowledge translation activities. In addition to smaller average cow-calf herd sizes in Eastern vs. Western Canada, several other differences were identified, some of which might be associated with differences in herd health and productivity. Herds from Eastern Canada were more likely to use reproductive technologies such as AI and also had longer breeding and calving seasons with calving seasons less likely to be concentrated in the early spring of the year. Herds from Eastern Canada were also more likely to report producer interventions and assistance at calving than herds from Western Canada.

When average regional differences were considered between herds from Eastern and Western Canada, most productivity and health outcomes were not significantly different. Calves were more likely to be treated for diarrhea from birth to 30 days and birth to weaning and more likely to die from calf diarrhea to both 30 days and to weaning in Eastern Canada than in Western Canada. While calf death loss due to navel infection was less frequent than due to either respiratory disease or calf diarrhea, death due to navel infection from birth to 30 days was also more common in Eastern than Western Canada.

In addition to questions about the impact of regional differences in management practices on productivity, the WCCCSN had found the timing of the start of breeding and calving season was a consistent source of variation in risks of non-pregnancy and calf losses (19). The use of large pastures for calving and the potential for links to the relative intensity of calving management was also identified as an important risk factor for some outcomes. Based on these findings, herds in the C3SN were categorized based on when they started to calve and whether they used large pastures for calving as an indicator of relative intensity of environmental contamination, disease transmission risk, and ability to observe calving and calf health. In the present study, herds calving earlier reported more calves treated for respiratory disease, diarrhea, and navel or joint infection and more calf deaths due to respiratory disease than herds that calved later. In a previous study from Western Canada, herds calving between December and February had a higher risk of respiratory disease compared to herds calving in March; however, the risk in that study was also lower for herds calving in March as compared to April and May (25). Notably, this study included data from only 1 year, and the April and May outbreaks were attributed to severe late spring snow storms highlighting the value of the new multiyear data. Another large cross-sectional study from the United States also reported an increased risk of respiratory disease for herds calving in the winter (27).

In the present study, the use of larger pastures for calving was associated with fewer calves treated for navel or joint infection, and calves in herds from the west had a lower risk of treatment and death from diarrhea than herds from the east where barn use and shelters are more common. The risks associated with not using large pastures was consistent with a recent paper examining data from the WCCCSN (25) that showed using high-density calving areas, such as pens or barns vs. pasture, or failure to use the Sandhills or similar calving management systems were associated with higher risks of calf diarrhea in specific age groups and navel ill. Previous reports also describe the impact of more crowded conditions on opportunities for direct transmission from infected animals or indirect transmission through environmental contamination and the associated value of the Sandhills calving management system (28, 29).

Similar to the WCCCSN analysis, herds that calved later with a later breeding season were also more likely to have more females that were not pregnant at testing in the fall (19). In the analysis of the 2014 to 2017 pregnancy test data, cows from herds in which the breeding season did not start until July and August were at significantly higher risk of non-pregnancy. The proposed most plausible explanation is the challenge of fulfilling the cow’s needed plane of nutrition for recovering from calving, early lactation, and starting cycle in later stages of the growing season and lower pasture quality, particularly in the face of recent droughts in Western Canada. For the current analysis, herds starting to calve between April and September were classified as calving later. The subsequent breeding season for 80% of the cow herd records was reported to start in July or August, closely matching data from the previous study.

The C3SN has been successful because of the participating cow-calf producers. The network relies on interested producers rather than attempting to source data from a random sample of Canadian cow-calf herds. For several reasons previously documented (19), random sampling is not a practical option for recruitment of herds for a long-term commitment to a surveillance network. The choice to recruit herds using the same approaches as previous studies in Western Canada facilitated comparison of the current results to historical data (19, 21–23, 30). The herds that participated in this network also include those committed to the cow-calf industry and those very likely to be influencers based on the importance of the cow-calf operation to their farm income, their management practices, and other herd and management attributes. The diffusion model of knowledge translation suggests that targeting early adopters can be critical for identifying management practices with the most potential for uptake by industry (31, 32).

While the use of questionnaires to collect health and productivity data has been criticized for the quality of the resulting information and bias, most participants in the present study had access to herd and individual animal records that could be used to inform questionnaire responses. Questionnaires on productivity data were in hand and reminders to complete questionnaires were timed for each herd with the completion of calving or weaning to reduce the potential for recall bias. Herd owners were encouraged to consult their records and logical checks were completed on the data prior to analysis.

Related to the challenges of collecting data with questionnaires was the unavailability of complete data for some herds. While almost all herds typically conducted pregnancy testing, as reported at the time of recruitment, not all tested all cows. In some cases, typical pregnancy testing practices were disrupted by extreme weather events resulting in cattle being pastured in atypical locations. In most cases, the herds that did not test all females simply did not check cows destined to be culled. In a previous study, investigators covered the cost of pregnancy testing to reduce the likelihood this would happen (30). However, the difference in the present study between records from herds that did and did not report testing all females was minimal.

While the primary objective was to get the best data possible from a committed group of participants, the investigators were also interested in how the resulting data reflected the Canadian cow-calf industry. Publicly available Statistics Canada Census of Agriculture data (33) were compared to the distribution of herds by province and herd sizes. The herd sizes included in this study represent the cows from more than 75% of all beef herds in Canada. Within Western Canada, the proportion of herds from each province was reasonably reflective of the 2021 Agriculture Census data for the number of beef cows: Alberta 44%, Saskatchewan 30%, Manitoba 11%, and British Columbia 5%. The proportion of herds from Eastern Canada in the present study was higher than what would reflect the distribution of beef cow-calf herds in Canada: Ontario 6%, Quebec 3%, and the Maritimes 1%. The decision was made to deliberately recruit additional herds from these regions to provide more power to describe practices in Eastern Canadian herds.

In summary, the unique longitudinal productivity and health data resulting from this network build on previous studies and provide a unique national baseline on which to build future research. Examples of research questions published to date from this network include addressing questions on Johne’s disease, trace mineral nutrition, vaccination practices, antimicrobial use and resistance (10, 26, 34–40). This information will inform region-specific needs for knowledge translation and solutions to enhance productivity and support sustainability.

## Data availability statement

The datasets presented in this article are not readily available because the data sharing agreement with the herd owners included a clause that only summarized data would be publicly shared. Detailed distributions of all relevant data are provided in tables within the paper and supplemental tables. Requests to access the datasets should be directed to cheryl.waldner@usask.ca.

## Ethics statement

The animal studies were approved by Animal Care Committee and Research Ethics Board (Protocol # 2014003) and the Behavioral Research Ethics Board (Beh-REB#309) at the University of Saskatchewan and the Comité d’éthique de la recherche en sciences et en santé (# CERSES-19-016-D) of the Université de Montréal. The studies were conducted in accordance with the local legislation and institutional requirements. Written informed consent was obtained from the owners for the participation of their animals in this study.

## Author contributions

CW: Conceptualization, Data curation, Formal analysis, Funding acquisition, Methodology, Project administration, Resources, Supervision, Validation, Writing – original draft, Writing – review & editing. MW: Methodology, Writing – review & editing. MR: Methodology, Writing – review & editing. JC: Conceptualization, Funding acquisition, Methodology, Project administration, Supervision, Writing – review & editing, Resources.
