# Supplementary material for: The Canadian Cow-Calf Surveillance Network – productivity and health summary 2018 to 2022
Source: Front Vet Sci. 2024 Apr 10;11:1392166. doi: 10.3389/fvets.2024.1392166 (PMC11040676; doi:10.3389/fvets.2024.1392166)
Supplement: Supplementary file 1 [file Table_1.pdf]

Supplemental table 1:

The Canadian Cow-calf Surveillance Network – Productivity and Health Data 2018 to 2022

Cheryl Waldner[1]*, M. Claire Windeyer[2], Marjolaine Rousseau[3], John Campbell[1]

[1]Large Animal Clinical Sciences, University of Saskatchewan, Saskatoon, SK, Canada

[2]Faculty of Veterinary Medicine, University of Calgary, Calgary, AB, Canada

[3]Département de sciences cliniques, Faculté de médecine vétérinaire, Université de Montréal, Saint-Hyacinthe, QC, Canada

Table S1. Summary of cattle purchases between weaning the previous year and the start of breeding season as well as the percentage of the herd that is purebred from Canadian cow-calf herds reported in submitted annual herd calving records (n=565) for the C3SN between 2019 and 2022.

	Cattle purchased between last weaning date the previous year and before turn-out date for breeding in current year					Percentage of herd that is purebred
	Number of calves purchased	Number of cow-calf pairs purchased	Number of cows purchased	Number of heifers purchased	Number of bulls purchased	
Total herd records	N = 565	N = 565	N = 565	N = 565	N = 565	N = 565
Mean	1	0	3	5	2	17
SD*	7	2	10	20	2	31
2.5th percentile	0	0	0	0	0	0
5th percentile	0	0	0	0	0	0
25th percentile	0	0	0	0	0	0
Median	0	0	0	0	1	0
75th percentile	0	0	0	2	3	20
95th percentile	1	0	16	25	6	99
97.5th percentile	3	3	36	40	8	100

*Standard deviation
