# Supplementary material for: The Canadian Cow-Calf Surveillance Network – productivity and health summary 2018 to 2022
Source: Front Vet Sci. 2024 Apr 10;11:1392166. doi: 10.3389/fvets.2024.1392166 (PMC11040676; doi:10.3389/fvets.2024.1392166)
Supplement: Supplementary file 2 [file Table_2.pdf]

**Supplemental table 2:**

# The Canadian Cow-calf Surveillance Network – Productivity and Health Data 2018 to 2022

**Cheryl Waldner[1]*, M. Claire Windeyer[2], Marjolaine Rousseau[3], John Campbell[1]**

[1]Large Animal Clinical Sciences, University of Saskatchewan, Saskatoon, SK, Canada

[2]Faculty of Veterinary Medicine, University of Calgary, Calgary, AB, Canada

[3]Département de sciences cliniques, Faculté de médecine vétérinaire, Université de Montréal, Saint-Hyacinthe, QC, Canada

**Table S2.** Summary of cattle purchases between start of breeding season and the last day of weaning from Canadian cow-calf herds reported in submitted breeding to weaning records (n=543) for the C3SN between 2019 and 2022.

| | Cattle purchased between the first day of breeding season and the last weaning date in the current year | | | | |
|---|---|---|---|---|---|
| | Number of calves purchased | Number of cow-calf pairs purchased | Number of cows purchased | Number of heifers purchased | Number of bulls purchased |
| Total herd records | N = 543 | N = 543 | N = 543 | N = 543 | N = 543 |
| Mean | 0 | 0 | 0 | 2 | 1 |
| SD* | 2 | 3 | 3 | 14 | 2 |
| 2.5$^{th}$ percentile | 0 | 0 | 0 | 0 | 0 |
| 5$^{th}$ percentile | 0 | 0 | 0 | 0 | 0 |
| 25$^{th}$ percentile | 0 | 0 | 0 | 0 | 0 |
| Median | 0 | 0 | 0 | 0 | 0 |
| 75$^{th}$ percentile | 0 | 0 | 0 | 0 | 1 |
| 95$^{th}$ percentile | 1 | 0 | 1 | 5 | 5 |
| 97.5$^{th}$ percentile | 2 | 1 | 2 | 13 | 6 |

*Standard deviation
