# Supplementary material for: The Canadian Cow-Calf Surveillance Network – productivity and health summary 2018 to 2022
Source: Front Vet Sci. 2024 Apr 10;11:1392166. doi: 10.3389/fvets.2024.1392166 (PMC11040676; doi:10.3389/fvets.2024.1392166)
Supplement: Supplementary file 3 [file Table_3.pdf]

Supplemental tables 3a, 3b:

The Canadian Cow-calf Surveillance Network – Productivity and Health Data 2018 to 2022

Cheryl Waldner[1]*, M. Claire Windeyer[2], Marjolaine Rousseau[3], John Campbell[1]

[1]Large Animal Clinical Sciences, University of Saskatchewan, Saskatoon, SK, Canada

[2]Faculty of Veterinary Medicine, University of Calgary, Calgary, AB, Canada

[3]Département de sciences cliniques, Faculté de médecine vétérinaire, Université de Montréal, Saint-Hyacinthe, QC, Canada

Table S3a. Summary of pregnancy outcome indicators from **Western Canadian** cow-calf herds reported in submitted annual herd calving records (n=379) for the C3SN between 2019 and 2022.

	Number calving			Percent of pregnant cows that aborted			Percent of calves dead with 24 h			Percent of calving females with twins		
	Cows	Heifers	Total	Cows	Heifers	Total	Cows	Heifers	Total	Cows	Heifers	Total
Total herd records	N=379	N=357	N=379	N=373	N=355	N=373	N=378	N=358	N=378	N=379	N=358	N=379
Mean	206	42	245	1.5%	3.0%	1.8%	2.1%	3.7%	2.4%	2.8%	1.3%	2.5%
SD*	162	38	193	1.7%	9.4%	2.7%	1.7%	4.3%	1.7%	2.0%	2.4%	1.8%
2.5th percentile	37	8	48	0.0%	0.0%	0.0%	0.0%	0.0%	0.0%	0.0%	0.0%	0.0%
5th percentile	48	9	60	0.0%	0.0%	0.0%	0.0%	0.0%	0.0%	0.0%	0.0%	0.0%
25th percentile	101	18	121	0.6%	0.0%	0.7%	1.0%	0.0%	1.2%	0.0%	0.0%	0.0%
Median	169	32	200	1.1%	0.0%	1.3%	1.8%	2.8%	2.1%	1.2%	0.0%	1.3%
75th percentile	263	53	311	2.1%	3.5%	2.2%	3.1%	5.7%	3.3%	2.4%	0.0%	2.2%
95th percentile	470	103	589	4.4%	9.2%	4.5%	5.3%	11.1%	5.7%	3.7%	2.0%	3.4%
97.5th percentile	754	149	885	5.7%	12.9%	6.5%	6.0%	14.3%	6.2%	6.7%	6.7%	5.9%

*Standard deviation

Table S3b. Summary of pregnancy outcome indicators from **Eastern Canadian** cow-calf herds reported in submitted annual herd calving records (n=186) for the C3SN between 2019 and 2022.

	Number calving			Percent of pregnant cows that aborted			Percent of calves dead with 24 h			Percent of calving females with twins		
	Cows	Heifers	Total	Cows	Heifers	Total	Cows	Heifers	Total	Cows	Heifers	Total
Total herd records	N=186	N=163	N=186	N=186	N=163	N=186	N=185	N=164	N=185	N=185	N=165	N=185
Mean	83	14	95	1.6%	2.6%	1.7%	3.3%	4.4%	3.4%	3.9%	1.5%	3.6%
SD*	75	16	88	2.3%	9.2%	2.3%	3.0%	8.0%	2.9%	3.4%	4.8%	3.1%
2.5th percentile	23	1	26	0.0%	0.0%	0.0%	0.0%	0.0%	0.0%	0.0%	0.0%	0.0%
5th percentile	27	3	30	0.0%	0.0%	0.0%	0.0%	0.0%	0.0%	0.0%	0.0%	0.0%
25th percentile	39	6	45	0.0%	0.0%	0.0%	1.1%	0.0%	1.3%	1.6%	0.0%	1.5%
Median	63	10	73	1.0%	0.0%	1.1%	2.7%	0.0%	2.7%	3.1%	0.0%	2.9%
75th percentile	91	16	104	2.5%	0.0%	2.5%	4.8%	6.7%	4.7%	5.1%	0.0%	4.8%
95th percentile	223	45	254	5.4%	14.1%	5.1%	9.4%	20.0%	9.3%	11.0%	9.1%	9.0%
97.5th percentile	272	50	290	7.4%	24.6%	8.3%	10.8%	25.0%	10.4%	11.8%	12.4%	10.8%

*Standard deviation
