# Supplementary material for: The Canadian Cow-Calf Surveillance Network – productivity and health summary 2018 to 2022
Source: Front Vet Sci. 2024 Apr 10;11:1392166. doi: 10.3389/fvets.2024.1392166 (PMC11040676; doi:10.3389/fvets.2024.1392166)
Supplement: Supplementary file 4 [file Table_4.pdf]

Supplemental tables 4a, 4b:

The Canadian Cow-calf Surveillance Network – Productivity and Health Data 2018 to 2022

Cheryl Waldner[1]*, M. Claire Windeyer[2], Marjolaine Rousseau[3], John Campbell[1]

[1]Large Animal Clinical Sciences, University of Saskatchewan, Saskatoon, SK, Canada

[2]Faculty of Veterinary Medicine, University of Calgary, Calgary, AB, Canada

[3]Département de sciences cliniques, Faculté de médecine vétérinaire, Université de Montréal, Saint-Hyacinthe, QC, Canada

Table S4a. Summary of calving difficulty outcomes from **Western Canadian** cow-calf herds reported in submitted annual herd calving records (n=379) for the C3SN between 2019 and 2022.

	Percent of females calved assisted			Percent of females with easy pull*			Percent of females with hard pull**			Percent of females with Caesarean section		
	Cows	Heifers	Total	Cows	Heifers	Total	Cows	Heifers	Total	Cows	Heifers	Total
Total herd records	N=379	N=359	N=379	N=377	N=377	N=357	N=377	N=357	N=379	N=376	N=358	N=379
Mean	4.5%	16.2%	6.4%	2.7%	9.3%	3.8%	1.4%	6.1%	2.1%	0.2%	0.9%	0.3%
SD***	7.0%	14.3%	7.6%	4.0%	10.2%	4.7%	2.1%	8.0%	2.7%	0.4%	2.4%	0.6%
2.5th percentile	0.0%	0.0%	0.0%	0.0%	0.0%	0.0%	0.0%	0.0%	0.0%	0.0%	0.0%	0.0%
5th percentile	0.0%	0.0%	0.0%	0.0%	0.0%	0.0%	0.0%	0.0%	0.0%	0.0%	0.0%	0.0%
25th percentile	0.9%	5.4%	2.1%	0.0%	0.5%	1.0%	0.0%	0.0%	0.4%	0.0%	0.0%	0.0%
Median	2.3%	12.5%	4.1%	1.2%	6.7%	2.3%	0.7%	3.3%	1.2%	0.0%	0.0%	0.0%
75th percentile	5.5%	23.6%	8.4%	3.3%	13.0%	4.9%	1.7%	9.1%	2.8%	0.0%	0.0%	0.3%
95th percentile	14.3%	44.5%	19.0%	10.5%	29.4%	12.2%	6.2%	21.9%	7.5%	1.0%	6.4%	1.5%
97.5th percentile	20.0%	50.9%	24.4%	13.3%	36.9%	14.4%	7.9%	28.7%	9.5%	1.3%	8.0%	2.0%

* one person only assist

** two person assist or required mechanical aid (e.g., calf jack)

***Standard deviation

Table S4b. Summary of calving difficulty outcomes from **Eastern Canadian** cow-calf herds reported in submitted annual herd calving records (n=186) for the C3SN between 2019 and 2022.

	Percent of females calved assisted			Percent of females with easy pull*			Percent of females with hard pull**			Percent with Caesarean section		
	Cows	Heifers	Total	Cows	Heifers	Total	Cows	Heifers	Total	Cows	Heifers	Total
Total herd records	N=183	N=163	N=183	N=182	N=162	N=182	N=183	N=163	N=183	N=182	N=164	N=182
Mean	7.4%	24.4%	9.5%	5.8%	12.3%	6.6%	3.0%	13.3%	4.2%	0.1%	0.1%	0.1%
SD***	8.8%	24.9%	10.0%	12.5%	16.7%	12.1%	4.1%	19.7%	5.0%	0.4%	0.6%	0.4%
2.5th percentile	0.0%	0.0%	0.0%	0.0%	0.0%	0.0%	0.0%	0.0%	0.0%	0.0%	0.0%	0.0%
5th percentile	0.0%	0.0%	0.0%	0.0%	0.0%	0.0%	0.0%	0.0%	0.0%	0.0%	0.0%	0.0%
25th percentile	1.7%	2.7%	2.5%	0.0%	0.0%	0.7%	0.0%	0.0%	0.9%	0.0%	0.0%	0.0%
Median	4.4%	18.8%	6.7%	2.1%	5.9%	2.9%	1.6%	6.7%	2.3%	0.0%	0.0%	0.0%
75th percentile	9.5%	37.5%	13.2%	6.6%	19.5%	7.4%	4.0%	20.0%	5.9%	0.0%	0.0%	0.0%
95th percentile	24.9%	79.3%	27.7%	20.5%	50.0%	21.5%	11.6%	50.0%	14.8%	0.0%	0.0%	0.3%
97.5th percentile	29.3%	90.5%	33.3%	43.4%	57.1%	47.3%	15.7%	54.5%	18.9%	1.6%	0.0%	1.4%

* one-person only assist

** two-person assist or required mechanical aid (e.g., calf jack)

***Standard deviation
