# Supplementary material for: The Canadian Cow-Calf Surveillance Network – productivity and health summary 2018 to 2022
Source: Front Vet Sci. 2024 Apr 10;11:1392166. doi: 10.3389/fvets.2024.1392166 (PMC11040676; doi:10.3389/fvets.2024.1392166)
Supplement: Supplementary file 5 [file Table_5.pdf]

Supplemental tables 5a, 5b:

The Canadian Cow-calf Surveillance Network – Productivity and Health Data 2018 to 2022

Cheryl Waldner[1]*, M. Claire Windeyer[2], Marjolaine Rousseau[3], John Campbell[1]

[1]Large Animal Clinical Sciences, University of Saskatchewan, Saskatoon, SK, Canada

[2]Faculty of Veterinary Medicine, University of Calgary, Calgary, AB, Canada

[3]Département de sciences cliniques, Faculté de médecine vétérinaire, Université de Montréal, Saint-Hyacinthe, QC, Canada

Table S5a. Summary of calving sickness and death loss from 24 hours to 30 days from **Western Canadian** cow-calf herds reported in submitted annual herd calving records (n=379) for the C3SN between 2019 and 2022.

	Percent of calves dead 24 h to 30 d			Percent of calves reported treated with antibiotics 24 h to 30 d			Percent of calves dead with attributed cause 24 h to 30 d			
	Cows	Heifers	Total	Calf diarrhea	Respiratory disease	Navel or joint infection	Calf diarrhea	Respiratory disease	Navel or joint infection	Total
Total herd records	N=378	N=356	N=378	N=376	N=374	N=374	N=375	N=375	N=375	N=375
Mean	1.6%	1.9%	1.7%	2.4%	2.8%	1.8%	0.4%	0.3%	0.1%	0.7%
SD*	1.6%	3.5%	1.6%	4.4%	5.0%	3.5%	0.9%	0.6%	0.3%	1.2%
2.5th percentile	0.0%	0.0%	0.0%	0.0%	0.0%	0.0%	0.0%	0.0%	0.0%	0.0%
5th percentile	0.0%	0.0%	0.0%	0.0%	0.0%	0.0%	0.0%	0.0%	0.0%	0.0%
25th percentile	0.5%	0.0%	0.6%	0.0%	0.0%	0.0%	0.0%	0.0%	0.0%	0.0%
Median	1.1%	0.0%	1.2%	0.9%	0.9%	0.6%	0.0%	0.0%	0.0%	0.3%
75th percentile	2.3%	2.6%	2.3%	2.6%	2.9%	1.8%	0.5%	0.4%	0.0%	0.9%
95th percentile	4.5%	9.6%	4.6%	9.4%	12.2%	8.3%	1.9%	1.4%	0.6%	3.1%
97.5th percentile	5.7%	11.1%	5.9%	16.1%	16.9%	11.5%	2.5%	1.8%	0.8%	4.1%

*Standard deviation

Table S5b. Summary of calving sickness and death loss from 24 hours to 30 days from **Eastern Canadian** cow-calf herds reported in submitted annual herd calving records (n=186) for the C3SN between 2019 and 2022.

	Percent of calves dead 24 h to 30 d			Percent of calves reported treated with antibiotics 24 h to 30 d			Percent of calves dead with attributed cause 24 h to 30 d			
	Cows	Heifers	Total	Calf diarrhea	Respiratory disease	Navel or joint infection	Calf diarrhea	Respiratory disease	Navel or joint infection	Total
Total herd records	N=184	N=165	N=184	N=181	N=184	N=182	N=183	N=183	N=183	N=183
Mean	2.3%	3.1%	2.4%	7.0%	3.7%	3.0%	0.7%	0.5%	0.2%	1.5%
SD*	2.8%	9.2%	2.9%	12.2%	6.8%	5.0%	1.8%	2.0%	1.1%	3.2%
2.5th percentile	0.0%	0.0%	0.0%	0.0%	0.0%	0.0%	0.0%	0.0%	0.0%	0.0%
5th percentile	0.0%	0.0%	0.0%	0.0%	0.0%	0.0%	0.0%	0.0%	0.0%	0.0%
25th percentile	0.0%	0.0%	0.0%	0.0%	0.0%	0.0%	0.0%	0.0%	0.0%	0.0%
Median	1.6%	0.0%	1.8%	3.4%	1.0%	1.0%	0.0%	0.0%	0.0%	0.0%
75th percentile	2.9%	0.0%	3.4%	9.1%	4.7%	4.0%	0.5%	0.0%	0.0%	2.0%
95th percentile	6.6%	14.3%	7.7%	23.5%	15.2%	14.2%	3.8%	2.5%	1.0%	7.1%
97.5th percentile	10.8%	19.7%	9.8%	42.1%	21.1%	17.9%	5.7%	3.4%	1.9%	9.9%

*Standard deviation
