# Supplementary material for: The Canadian Cow-Calf Surveillance Network – productivity and health summary 2018 to 2022
Source: Front Vet Sci. 2024 Apr 10;11:1392166. doi: 10.3389/fvets.2024.1392166 (PMC11040676; doi:10.3389/fvets.2024.1392166)
Supplement: Supplementary file 6 [file Table_6.pdf]

Supplemental tables 6a, 6b:

The Canadian Cow-calf Surveillance Network – Productivity and Health Data 2018 to 2022

Cheryl Waldner[1]*, M. Claire Windeyer[2], Marjolaine Rousseau[3], John Campbell[1]

[1]Large Animal Clinical Sciences, University of Saskatchewan, Saskatoon, SK, Canada

[2]Faculty of Veterinary Medicine, University of Calgary, Calgary, AB, Canada

[3]Département de sciences cliniques, Faculté de médecine vétérinaire, Université de Montréal, Saint-Hyacinthe, QC, Canada

Table S6a. Summary of breeding stock and female to bull ratios for **Western Canadian** cow-calf herds reported in submitted annual herd breeding to weaning records (n=364) for the C3SN between 2019 and 2022.

	Bulls bred to cows	Bulls bred to heifers	Cows exposed to breeding	Heifers exposed to breeding	Cow to bull ratio	Heifer to bull ratio
Total herd records	N=363	N=348	N=363	N=350	N=360	N=323
Mean	10	2	231	51	23	18
SD*	8	3	183	50	7	9
2.5th percentile	2	1	38	5	9	2
5th percentile	2	1	54	10	13	3
25th percentile	5	1	115	20	19	12
Median	8	2	189	35	22	18
75th percentile	12	3	292	63	27	24
95th percentile	25	5	556	136	34	33
97.5th percentile	40	6	882	208	36	36

*Standard deviation

Table S6b. Summary of breeding stock and female to bull ratios for **Eastern Canadian** cow-calf herds reported in submitted annual herd breeding to weaning records (n=179) for the C3SN between 2019 and 2022.

	Bulls bred to cows	Bulls bred to heifers	Cows exposed to breeding	Heifers exposed to breeding	Cow to bull ratio	Heifer to bull ratio
Total herd records	N=179	N=164	N=179	N=167	N=175	N=125
Mean	4	1	90	15	24	12
SD*	4	1	76	19	13	13
2.5th percentile	1	0	18	0	4	1
5th percentile	1	0	32	1	6	1
25th percentile	2	1	46	6	15	5
Median	3	1	71	10	22	8
75th percentile	4	1	103	16	28	14
95th percentile	11	3	236	50	47	34
97.5th percentile	12	3	281	70	50	56

*Standard deviation
