# Supplementary material for: The Canadian Cow-Calf Surveillance Network – productivity and health summary 2018 to 2022
Source: Front Vet Sci. 2024 Apr 10;11:1392166. doi: 10.3389/fvets.2024.1392166 (PMC11040676; doi:10.3389/fvets.2024.1392166)
Supplement: Supplementary file 7 [file Table_7.pdf]

Supplemental tables 7a, 7b:

The Canadian Cow-calf Surveillance Network – Productivity and Health Data 2018 to 2022

Cheryl Waldner[1]*, M. Claire Windeyer[2], Marjolaine Rousseau[3], John Campbell[1]

[1]Large Animal Clinical Sciences, University of Saskatchewan, Saskatoon, SK, Canada

[2]Faculty of Veterinary Medicine, University of Calgary, Calgary, AB, Canada

[3]Département de sciences cliniques, Faculté de médecine vétérinaire, Université de Montréal, Saint-Hyacinthe, QC, Canada

Table S7a. Summary of percentage of females bred using reproductive technologies and breeding season duration for **Western Canadian** cow-calf herds reported in submitted annual herd breeding to weaning records (n=364) for the C3SN between 2019 and 2022.

	Percent of cows bred AI/ET	Percent of heifers bred AI/ET	Breeding season length cows (days)	Breeding season length heifers (days)
Total herd records	N=363	N=347	N=357	N=338
Mean	6.4%	17.2%	85	78
SD*	16.5%	33.1%	35	37
2.5th percentile	0.0%	0.0%	45	25
5th percentile	0.0%	0.0%	48	30
25th percentile	0.0%	0.0%	61	50
Median	0.0%	0.0%	74	68
75th percentile	0.0%	5.3%	99	98
95th percentile	47.3%	99.5%	162	146
97.5th percentile	60.9%	100%	173	165

*Standard deviation

Table S7b. Summary of percentage of females bred using reproductive technologies and breeding season duration for **Eastern Canadian** cow-calf herds reported in submitted annual herd breeding to weaning records (n=179) for the C3SN between 2019 and 2022.

	Percent of cows bred AI/ET	Percent of heifers bred AI/ET	Breeding season length cows (days)	Breeding season length heifers (days)
Total herd records	N=179	N=159	N=162	N=154
Mean	23.6%	37.5%	136	116
SD*	31.2%	42.5%	70	58
2.5th percentile	179	159	53	44
5th percentile	23.6%	37.5%	57	47
25th percentile	31.2%	42.5%	80	72
Median	0.0%	0.0%	114	102
75th percentile	0.0%	0.0%	184	146
95th percentile	0.0%	0.0%	259	233
97.5th percentile	7.3%	8.3%	291	262

*Standard deviation
