# Supplementary material for: The Canadian Cow-Calf Surveillance Network – productivity and health summary 2018 to 2022
Source: Front Vet Sci. 2024 Apr 10;11:1392166. doi: 10.3389/fvets.2024.1392166 (PMC11040676; doi:10.3389/fvets.2024.1392166)
Supplement: Supplementary file 8 [file Table_8.pdf]

Supplemental tables 8a, 8b, 8c:

The Canadian Cow-calf Surveillance Network – Productivity and Health Data 2018 to 2022

Cheryl Waldner[1]*, M. Claire Windeyer[2], Marjolaine Rousseau[3], John Campbell[1]

[1]Large Animal Clinical Sciences, University of Saskatchewan, Saskatoon, SK, Canada

[2]Faculty of Veterinary Medicine, University of Calgary, Calgary, AB, Canada

[3]Département de sciences cliniques, Faculté de médecine vétérinaire, Université de Montréal, Saint-Hyacinthe, QC, Canada

Table S8a. Summary of number of females pregnancy tested by a veterinarian and percentage of tested cows that were not pregnant for **Western Canadian** cow-calf herds reported in submitted annual herd breeding to weaning records (n=364) for the C3SN between 2019 and 2022.

	Number of cows pregnancy tested	Number of heifers pregnancy tested	Number of all females pregnancy tested	Percent of cows not pregnant	Percent of heifers not pregnant	Percent of all females not pregnant
Total herd records	N=341	N=334	N=341	N=341	N=334	N=341
Mean	227	50	274	7.5%	10.6%	8.2%
SD*	184	49	223	4.8%	10.0%	5.0%
2.5th percentile	32	6	48	0.0%	0.0%	1.3%
5th percentile	45	10	64	1.4%	0.0%	2.2%
25th percentile	111	21	130	4.1%	4.0%	4.5%
Median	177	35	216	6.8%	8.3%	7.2%
75th percentile	287	60	340	9.9%	14.2%	10.6%
95th percentile	585	129	711	16.8%	31.5%	17.3%
97.5th percentile	858	197	998	18.3%	37.4%	20.3%

*Standard deviation

Table S8b. Summary of number of females pregnancy tested by a veterinarian and percentage of tested cows that were not pregnant for **Eastern Canadian** cow-calf herds reported in submitted annual herd breeding to weaning records (n=179) for the C3SN between 2019 and 2022.

	Number of cows pregnancy tested	Number of heifers pregnancy tested	Number of all females pregnancy tested	Percent of cows not pregnant	Percent of heifers not pregnant	Percent of all females not pregnant
Total herd records	N=166	N=149	N=166	N=166	N=149	N=166
Mean	82	18	97	8.0%	8.0%	8.1%
SD*	85	22	100	7.1%	13.4%	6.6%
2.5th percentile	9	2	11	0.0%	0.0%	0.0%
5th percentile	10	2	17	0.0%	0.0%	1.0%
25th percentile	41	7	49	3.5%	0.0%	3.8%
Median	66	11	80	5.9%	0.0%	5.9%
75th percentile	90	18	99	10.5%	10.0%	10.2%
95th percentile	232	67	264	20.0%	35.3%	20.8%
97.5th percentile	278	86	297	24.0%	50.0%	24.8%

*Standard deviation

Table S8c. Summary of number of females pregnancy tested by a veterinarian and percentage of tested females that were not pregnant for Canadian cow-calf herds reported in submitted annual herd breeding to weaning records (n=543) for the C3SN between 2019 and 2022 (**including only herds that reported all females were pregnancy tested**).

	Number of cows pregnancy tested	Number of heifers pregnancy tested	Number of all females pregnancy tested	Percent of cows not pregnant	Percent of heifers not pregnant	Percent of all females not pregnant
Total herd records	N = 442	N = 418	N = 442	N = 442	N = 418	N = 442
Mean	189	40	226	7.6%	10.1%	8.1%
SD*	178	47	217	5.0%	11.2%	5.2%
2.5th percentile	31	3	38	0.8%	0.0%	1.2%
5th percentile	36	5	44	1.4%	0.0%	2.0%
25th percentile	72	12	84	4.0%	0.0%	4.3%
Median	131	25	160	6.5%	7.1%	6.9%
75th percentile	245	51	291	10.0%	14.3%	10.8%
95th percentile	465	126	644	16.9%	33.3%	19.1%
97.5th percentile	779	159	870	18.8%	40.1%	20.8%

*Standard deviation
