# Supplementary material for: The Canadian Cow-Calf Surveillance Network – productivity and health summary 2018 to 2022
Source: Front Vet Sci. 2024 Apr 10;11:1392166. doi: 10.3389/fvets.2024.1392166 (PMC11040676; doi:10.3389/fvets.2024.1392166)
Supplement: Supplementary file 9 [file Table_9.pdf]

Supplemental tables 9a, 9b:

The Canadian Cow-calf Surveillance Network – Productivity and Health Data 2018 to 2022

Cheryl Waldner[1]*, M. Claire Windeyer[2], Marjolaine Rousseau[3], John Campbell[1]

[1]Large Animal Clinical Sciences, University of Saskatchewan, Saskatoon, SK, Canada

[2]Faculty of Veterinary Medicine, University of Calgary, Calgary, AB, Canada

[3]Département de sciences cliniques, Faculté de médecine vétérinaire, Université de Montréal, Saint-Hyacinthe, QC, Canada

Table S9a. Summary of calving death loss from 24 hours to weaning from **Western Canadian** cow-calf herds reported in submitted breeding to weaning records (n=364) for the C3SN between 2019 and 2022**.**

	Calves alive at 24 hours			Percent of calves dead 24 h to weaning		
	Cows	Heifers	Total	Cows	Heifers	Total
Total herd records	N=364	N=348	N=364	N=364	N=329	N=364
Mean	212	39	249	3.4%	4.9%	3.6%
SD*	207	37	231	3.1%	7.7%	3.1%
2.5th percentile	38	0	45	0.0%	0.0%	0.0%
5th percentile	45	3	57	0.0%	0.0%	0.0%
25th percentile	102	15	125	1.5%	0.0%	1.6%
Median	168	29	200	2.7%	2.9%	2.9%
75th percentile	261	49	315	4.4%	6.9%	4.7%
95th percentile	456	102	565	9.4%	17.6%	9.6%
97.5th percentile	766	147	870	11.3%	20.0%	11.6%

*Standard deviation

Table S9b. Summary of calving death loss from 24 hours to weaning from **Eastern Canadian** cow-calf herds reported in submitted breeding to weaning records (n=179) for the C3SN between 2019 and 2022**.**

	Calves alive at 24 hours			Percent of calves dead 24 h to weaning		
	Cows	Heifers	Total	Cows	Heifers	Total
Total herd records	N=179	N=166	N=179	N=178	N=152	N=178
Mean	84	12	96	4.7%	6.9%	4.9%
SD*	78	16	89	5.6%	10.6%	5.2%
2.5th percentile	23	0	25	0.0%	0.0%	0.0%
5th percentile	28	0	30	0.0%	0.0%	0.0%
25th percentile	40	5	46	1.5%	0.0%	1.9%
Median	66	9	77	3.4%	0.0%	3.7%
75th percentile	92	14	105	5.9%	10.1%	6.5%
95th percentile	237	33	260	14.1%	33.3%	13.9%
97.5th percentile	281	50	289	18.4%	34.0%	18.5%

*Standard deviation
