# Supplementary material for: The Canadian Cow-Calf Surveillance Network – productivity and health summary 2018 to 2022
Source: Front Vet Sci. 2024 Apr 10;11:1392166. doi: 10.3389/fvets.2024.1392166 (PMC11040676; doi:10.3389/fvets.2024.1392166)
Supplement: Supplementary file 10 [file Table_10.pdf]

Supplemental tables 10a, 10b:

The Canadian Cow-calf Surveillance Network – Productivity and Health Data 2018 to 2022

Cheryl Waldner[1]*, M. Claire Windeyer[2], Marjolaine Rousseau[3], John Campbell[1]

[1]Large Animal Clinical Sciences, University of Saskatchewan, Saskatoon, SK, Canada

[2]Faculty of Veterinary Medicine, University of Calgary, Calgary, AB, Canada

[3]Département de sciences cliniques, Faculté de médecine vétérinaire, Université de Montréal, Saint-Hyacinthe, QC, Canada

Table S10a. Summary of calf treatment and death loss for diarrhea, respiratory disease, and navel or joint infection from 24 hours to weaning from **Western Canadian** cow-calf herds reported in submitted breeding to weaning records (n=364) for the C3SN between 2019 and 2022**.**

	Percent of calves reported treated with antibiotics 24 h to weaning			Percent of calves dead with attributed cause 24 h to weaning			
	Calf diarrhea	Respiratory disease	Navel or joint infection	Calf diarrhea	Respiratory disease	Navel or joint infection	Total
Total herd records	N=362	N=362	N=362	N=362	N=362	N=362	N=362
Mean	2.3%	4.6%	1.8%	0.40%	0.57%	0.13%	1.1%
SD*	4.3%	8.8%	3.4%	1.1%	1.0%	0.36%	1.8%
2.5th percentile	0.0%	0.0%	0.0%	0.0%	0.0%	0.0%	0.0%
5th percentile	0.0%	0.0%	0.0%	0.0%	0.0%	0.0%	0.0%
25th percentile	0.0%	0.3%	0.0%	0.0%	0.0%	0.0%	0.0%
Median	0.7%	1.7%	0.5%	0.0%	0.0%	0.0%	0.47%
75th percentile	2.8%	4.7%	2.0%	0.42%	0.78%	0.0%	1.6%
95th percentile	9.2%	19.0%	7.8%	1.8%	2.5%	0.91%	4.6%
97.5th percentile	15.4%	26.8%	10.3%	2.7%	3.7%	1.3%	6.1%

*Standard deviation

Table S10b. Summary of calf treatment and death loss for diarrhea, respiratory disease, and navel or joint infection from 24 hours to weaning from **Eastern Canadian** cow-calf herds reported in submitted breeding to weaning records (n=364) for the C3SN between 2019 and 2022**.**

	Percent of calves reported treated with antibiotics 24 h to weaning			Percent of calves dead with attributed cause 24 h to weaning			
	Calf diarrhea	Respiratory disease	Navel or joint infection	Calf diarrhea	Respiratory disease	Navel or joint infection	Total
Total herd records	N=179	N=179	N=179	N=179	N=179	N=179	N=179
Mean	8.1%	6.6%	2.9%	1.2%	1.1%	0.32%	2.6%
SD*	15.2%	18.0%	4.6%	2.8%	4.7%	1.5%	6.7%
2.5th percentile	0.0%	0.0%	0.0%	0.00%	0.00%	0.00%	0.00%
5th percentile	0.0%	0.0%	0.0%	0.00%	0.00%	0.00%	0.00%
25th percentile	0.0%	0.0%	0.0%	0.00%	0.00%	0.00%	0.00%
Median	3.3%	1.7%	1.1%	0.00%	0.00%	0.00%	0.33%
75th percentile	9.2%	6.1%	3.9%	1.2%	0.82%	0.00%	2.6%
95th percentile	30.0%	20.3%	12.7%	5.7%	3.0%	1.9%	9.9%
97.5th percentile	62.3%	36.9%	17.4%	7.5%	4.7%	2.8%	16.0%

*Standard deviation
