# Supplementary material for: The Canadian Cow-Calf Surveillance Network – productivity and health summary 2018 to 2022
Source: Front Vet Sci. 2024 Apr 10;11:1392166. doi: 10.3389/fvets.2024.1392166 (PMC11040676; doi:10.3389/fvets.2024.1392166)
Supplement: Supplementary file 11 [file Table_11.pdf]

Supplemental tables 11a, 11b:

The Canadian Cow-calf Surveillance Network – Productivity and Health Data 2018 to 2022

Cheryl Waldner[1]*, M. Claire Windeyer[2], Marjolaine Rousseau[3], John Campbell[1]

[1]Large Animal Clinical Sciences, University of Saskatchewan, Saskatoon, SK, Canada

[2]Faculty of Veterinary Medicine, University of Calgary, Calgary, AB, Canada

[3]Département de sciences cliniques, Faculté de médecine vétérinaire, Université de Montréal, Saint-Hyacinthe, QC, Canada

Table S11a. Reproductive indices for comparison from **herds from Western Canada** for 2019 to 2022 and 2014 to 2017 *(previously reported in (19) and reproduced with permission from open access source*).

Herd Summary Statistics	Percent of females not pregnant when tested (2014-2017)		Percent of females not pregnant when tested (2019-2022)		Abortion cumulative incidence (2014-2017)		Abortion cumulative incidence (2019-2022)	
	Cows	*Heifers*	Cows	Heifers	*Cows*	*Heifers*	Cows	Heifers
Mean	*6.8%*	*9.7%*	7.5%	10.5%	*0.8%*	*1.3%*	1.5%	3.0%
SD*	*3.4%*	*8.2%*	4.8%	9.8%	*0.9%*	*2.3%*	1.7%	9.4%
5th percentile	*2.2%*	*0.0%*	1.4%	0.0%	*0.0%*	*0.0%*	0.0%	0.0%
25th percentile	*4.3%*	*4.2%*	4.1%	4.2%	*0.0%*	*0.0%*	0.6%	0.0%
Median	*6.2%*	*8.3%*	6.8%	8.3%	*0.7%*	*0.0%*	1.1%	0.0%
75th percentile	*8.9%*	*12.9%*	9.9%	13.8%	*1.2%*	*2.1%*	2.1%	3.5%
95th percentile	*12.8%*	*24.1%*	16.8%	30.6%	*2.6%*	*5.6%*	4.4%	9.2%
Observations	*276*	*270*	340	318	*353*	*338*	373	355
Number of herds	*94*	*90*	108	106	*105*	*104*	112	110

*Standard deviation

Table S11b. Reproductive indices for comparison from **herds from Western Canada** for 2019 to 2022 and 2014 to 2017 (*previously reported (19) and reproduced with permission from open access source*).

Herd Summary Statistics	Cumulative incidence of calf death from birth – 24 hours (2014-2017)		Cumulative incidence of calf death from birth – 24 hours (2019-2022)		Cumulative incidence of calf death from 24 hours to weaning (2014-2017)		Cumulative incidence of calf death from 24 hours to weaning (2019-2022)	
	Cows	*Heifers*	Cows	Heifers	*Cows*	*Heifers*	Cows	Heifers
Mean	*2.1%*	*3.6%*	2.1%	3.7%	*2.5%*	*2.9%*	3.4%	4.9%
SD*	*1.6%*	*4.5%*	1.7%	4.3%	*2.4%*	*3.9%*	3.1%	7.7%
5th percentile	*0.0%*	*0.0%*	0.0%	0.0%	*0.0%*	*0.0%*	0.0%	0.0%
25th percentile	*1.0%*	*0.0%*	1.0%	0.0%	*1.1%*	*0.0%*	1.5%	0.0%
Median	*1.8%*	*2.6%*	1.8%	2.8%	*1.9%*	*1.5%*	2.7%	2.9%
75th percentile	*2.8%*	*5.3%*	3.1%	5.7%	*3.3%*	*4.6%*	4.4%	6.9%
95th percentile	*4.8%*	*11.5%*	5.3%	11.1%	*6.9%*	*10.4%*	9.4%	17.6%
Observations	*359*	*345*	378	358	*350*	*332*	364	329
Number of herds	*105*	*105*	114	112	*105*	*105*	112	108

*Standard deviation
