# Supplementary material for: The Canadian Cow-Calf Surveillance Network – productivity and health summary 2018 to 2022
Source: Front Vet Sci. 2024 Apr 10;11:1392166. doi: 10.3389/fvets.2024.1392166 (PMC11040676; doi:10.3389/fvets.2024.1392166)
Supplement: Supplementary file 12 [file Table_12.pdf]

Supplemental table 12:

The Canadian Cow-calf Surveillance Network – Productivity and Health Data 2018 to 2022

Cheryl Waldner[1]*, M. Claire Windeyer[2], Marjolaine Rousseau[3], John Campbell[1]

[1]Large Animal Clinical Sciences, University of Saskatchewan, Saskatoon, SK, Canada

[2]Faculty of Veterinary Medicine, University of Calgary, Calgary, AB, Canada

[3]Département de sciences cliniques, Faculté de médecine vétérinaire, Université de Montréal, Saint-Hyacinthe, QC, Canada

Table S12. Mean production indices (SD*) of **herds from Western Canada** (N) by year of testing 2019-2022 compared to data from 2014-2017 (*previously reported (19) and reproduced with permission from open access source*).

		2014	*2015*	*2016*	*2017*	**2019**	**2020**	**2021**	**2022**
Percent of females not pregnant at pregnancy testing	Cows	*6.7% (2.7) N=69*	*6.6% (3.9) N=75*	*6.8% (3.5) N=73*	*7.1% (3.7) N=59*	7.2% (4.5) N=109	7.1% (4.2) N=97	8.2% (5.8) N=82	7.7% (5.0) N=76
	Heifers	*9.1% (7.3) N=65*	*9.6% (8.5) N=75*	*10.1% (7.9) N=72*	*9.9% (9.1) N=58*	9.9% (8.5) N=109	10.1% (9.5) N=97	9.5% (8.7) N=82	13.2% (12.4) N=76
Abortion cumulative incidence	Cows	*1.0% (1.0) N=83*	*0.7% (0.7) N=93*	*0.8% (0.9) N=91*	*0.9% (0.8) N=86*	1.5% (1.4) N=113	1.6% (1.5) N=101	1.5% (1.5) N=85	1.6% (2.4) N=80
	Heifers	*1.6% (2.5) N=79*	*0.9% (1.9) N=90*	*1.5% (2.9) N=86*	*1.4% (2.0) N=83*	2.7% (8.4) N=113	3.2% (10.6) N=101	3.8% (12.2) N=85	2.3% (3.4) N=80
Cumulative incidence of calf death from birth – 24 hours	Cows	*2.4% (1.6) N=84*	*2.0% (1.4) N=93*	*1.9% (1.4) N=95*	*2.1% (1.9) N=87*	2.2% (1.9) N=113	2.1% (1.5) N=101	1.9% (1.4) N=85	2.3% (1.9) N=80
	Heifers	*4.7% (5.7) N=79*	*3.8% (4.3) N=90*	*3.0% (4.0) N=92*	*2.9% (3.8) N=84*	3.8% (4.5) N=113	3.9% (4.1) N=101	3.2% (4.7) N=85	3.6% (3.7) N=80
Cumulative incidence of calf death (24 hours-weaning)	Cows	*2.7% (2.8) N=84*	*2.4% (2.0) N=94*	*2.3% (1.8) N=93*	*2.9% (2.9) N=90*	3.7% (3.3) N=109	3.6% (3.5) N=97	2.8% (2.1) N=82	3.5% (3.1) N=76
	Heifers	*3.4% (4.7) N=78*	*2.1% (2.8) N=90*	*3.2% (4.1) N=90*	*2.8% (3.8) N=90*	6.1% (11.2) N=109	4.2% (5.2) N=97	4.0% (5.1) N=82	4.8% (6.4) N=76

*Standard deviation
