# Supplementary material for: The Canadian Cow-Calf Surveillance Network – productivity and health summary 2018 to 2022
Source: Front Vet Sci. 2024 Apr 10;11:1392166. doi: 10.3389/fvets.2024.1392166 (PMC11040676; doi:10.3389/fvets.2024.1392166)
Supplement: Supplementary file 13 [file Table_13.pdf]

Supplemental table 13:

The Canadian Cow-calf Surveillance Network – Productivity and Health Data 2018 to 2022

Cheryl Waldner[1]*, M. Claire Windeyer[2], Marjolaine Rousseau[3], John Campbell[1]

[1]Large Animal Clinical Sciences, University of Saskatchewan, Saskatoon, SK, Canada

[2]Faculty of Veterinary Medicine, University of Calgary, Calgary, AB, Canada

[3]Département de sciences cliniques, Faculté de médecine vétérinaire, Université de Montréal, Saint-Hyacinthe, QC, Canada

Table S13. Mean production indices (SD*) of **herds (N) from Eastern Canada** by year of testing 2019-2022.

		2019	2020	2021	2022
Percent of females not pregnant at pregnancy testing	Cows	8.5% (6.5) N=46	8.4% (4.9) N=40	6.6% (5.9) N=33	7.1% (6.5) N=28
	Heifers	14.0% (19.1) N=46	8.2% (11.4) N=40	5.0% (6.8) N=33	3.3% (4.1) N=28
Abortion cumulative incidence	Cows	2.3% (3.1) N=58	1.2% (1.8) N=51	1.3% (1.5) N=40	1.6% (2.1) N=37
	Heifers	2.1% (5.8) N=58	3.3% (6.3) N=51	0.9% (2.8) N=40	1.2% (3.3) N=37
Cumulative incidence of calf death from birth – 24 hours	Cows	3.8% (3.5) N=58	3.2% (2.8) N=51	2.9% (2.6) N=40	3.0% (2.9) N=36
	Heifers	3.9% (3.3) N=58	4.9% (2.5) N=51	3.9% (2.7) N=40	4.8% (3.1) N=36
Cumulative incidence of calf death (24 hours-weaning)	Cows	5.5% (7.9) N=58	4.3% (4.6) N=49	4.1% (3.7) N=39	4.6% (3.5) N=33
	Heifers	7.3% (9.7) N=58	7.6% (11.9) N=49	5.4% (10.5) N=39	6.9% (10.5) N=33

*Standard deviation
